# Subversion of selective autophagy for the biogenesis of tombusvirus replication organelles inhibits autophagy

**DOI:** 10.1371/journal.ppat.1012085

**Published:** 2024-03-14

**Authors:** Yuanrong Kang, Wenwu Lin, Peter D. Nagy

**Affiliations:** Department of Plant Pathology, University of Kentucky, Lexington, Kentucky, United States of America; University of California, Davis Genome Center, UNITED STATES

## Abstract

Elaborate viral replication organelles (VROs) are formed to support positive-strand RNA virus replication in infected cells. VRO formation requires subversion of intracellular membranes by viral replication proteins. Here, we showed that the key ATG8f autophagy protein and NBR1 selective autophagy receptor were co-opted by Tomato bushy stunt virus (TBSV) and the closely-related carnation Italian ringspot virus. Knockdown of ATG8f or NBR1 in plants led to reduced tombusvirus replication, suggesting pro-viral function for selective autophagy. BiFC and proximity-labeling experiments showed that the TBSV p33 replication protein interacted with ATG8f and NBR1 to recruit them to VROs. In addition, we observed that several core autophagy proteins, such as ATG1a, ATG4, ATG5, ATG101 and the plant-specific SH3P2 autophagy adaptor proteins were also re-localized to TBSV VROs, suggesting that TBSV hijacks the autophagy machinery in plant cells. We demonstrated that subversion of autophagy components facilitated the recruitment of VPS34 PI3 kinase and enrichment of phospholipids, such as phosphatidylethanolamine and PI3P phosphoinositide in the VRO membranes. Hijacking of autophagy components into TBSV VROs led to inhibition of autophagic flux. We also found that a fraction of the subverted ATG8f and NBR1 was sequestered in biomolecular condensates associated with VROs. We propose that the VRO-associated condensates trap those autophagy proteins, taking them away from the autophagy pathway. Overall, tombusviruses hijack selective autophagy to provide phospholipid-rich membranes for replication and to regulate the antiviral autophagic flux.

## Author summary

Positive-strand RNA viruses are important pathogens of plants, animals and humans. These viruses replicate inside the infected cells in virus-specific replication organelles (VROs). Similar to other viruses, Tomato bushy stunt virus (TBSV) has to co-opt numerous host proteins, subcellular organellar membranes and vesicles to build VROs. Here, the authors found that TBSV co-opted the autophagy machinery for pro-viral functions. Autophagy is a degradation pathway to recycle proteins, damaged organelles or destruct pathogens. However, TBSV recruited members of the autophagy pathway to VROs, sequestering them in biomolecular condensates, which were associated with VROs. This led to inhibition of autophagic flux, and protected TBSV from the antiviral autophagic degradation. Moreover, subversion of autophagy components facilitated the recruitment of VPS34 PI3 kinase and enrichment of phospholipids, such as phosphatidylethanolamine and PI3P phosphoinositide in the VRO membranes. In summary, tombusviruses hijacked autophagy to provide phospholipid-rich membranes for replication and to regulate the antiviral autophagy.

## Introduction

Positive-strand (+)RNA viruses infect and cause many diseases in eukaryotic organisms. (+)RNA viruses have small genomes and they have to co-opt numerous host factors to support their replication inside the infected cells. Virus replication depends on the biogenesis of viral replication organelles (VROs), which cluster many membrane-bound viral replicase complexes (VRCs) [1–8]. VRO biogenesis requires usurping various intracellular organelles, membrane deformation, new lipid biosynthesis, phospholipid and sterol transfer and co-opting vesicular trafficking [7,9–12]. The membranous VROs sequester the viral (+)RNA and viral and co-opted host proteins for efficient replication. In addition, VROs also protect the viral (+)RNA and the dsRNA replication intermediate from recognition and elimination by the host innate immune system [13–20]. Altogether, the VROs coordinate the viral replication process spatiotemporally [7,9,21–23].

Tomato bushy stunt virus (TBSV), which is a small (+)RNA virus of plants, is studied intensively to unravel the basic mechanism of viral RNA replication [24–27]. TBSV codes for two essential replication proteins, the p92 RdRp and the p33 replication protein, which is the master regulator of VRO assembly and viral (+)RNA recruitment into VRCs [28,29]. TBSV replicon (rep)RNA replicates in the surrogate host yeast (*Saccharomyces cerevisiae*) to a high level [25,30,31]. Yeast-based genome-wide and proteome-wide studies with TBSV led to the identification of numerous host factors co-opted for viral RNA replication and recombination [2,25,32–35]. Overall, TBSV depends on global phospholipid and sterol biosynthesis [36–38]. Formation of viral replicase complexes (VRCs) and activation of the viral-coded p92 RdRp requires phospatidylethanolamine (PE), phosphoinositides and sterols [28,37,39–45].

TBSV induces subcellular membrane proliferation and peroxisome aggregation in both yeast and plant cells. One of the characteristic features of TBSV infection is the formation of virus-induced membrane contact sites (vMCSs) [25,46–48]. vMCS forms between the hijacked subdomain in the ER and the peroxisome with the help of p33 replication protein and co-opted host proteins, such as oxystrerol-binding proteins, ER-resident Sac1 PI4P phosphatase and VAP proteins and Fis1 mitochondrial fission protein [43,48,49]. vMCS function is essential for the enrichment of sterols and phosphoinositides within VROs [48] to protect TBSV replication protein from proteasomal degradation [28,44]. In addition to subversion of peroxisomes by TBSV and mitochondria by the closely related carnation Italian ringspot virus (CIRV), these viruses also co-opt a subdomain of ER containing a SNARE complex, including the syntaxin18-like protein [50,51]. Moreover, tombusviruses hijack Rab5-positive endosomes, Rab1-positive COP II vesicles and the retromer tubular transport carriers. These co-opted vesicles provide membranes, lipids, and lipid synthesis and modification enzymes for VRO biogenesis [39,52–54].

Autophagy plays an important role in maintaining cellular homeostasis and also in defense against invading pathogens in plants and animals. Autophagy is initiated by autophagy-related proteins (ATGs) at the phagophore assembly site (PAS) by recruitment of cargo proteins or damaged organelles, followed by maturation into autophagosome [55–59]. The autophagosomes are double-membrane organelles, which deliver the cargoes into vacuoles for degradation and recycling. Recent studies revealed an important antiviral role of autophagy that leads to degradation of viral proteins or virions. However, several viruses block autophagy or utilize autophagy to degrade host defense factors, whereas other viruses exploit autophagy to support virus replication [60–68]. Autophagy also targets plant viruses and it is a major player in antiviral innate immunity [69–75]. Several plant viruses manipulate the autophagy machinery to inhibit antiviral defenses in plants [76–80]. However, plant viruses also exploit autophagy for pro-viral functions [65,81,82].

ATG8 is a ubiquitin-like protein that is essential for autophagy [83,84]. ATG8 is activated through sequential steps by several ATG proteins, ultimately leading to lipidation of ATG8. ATG8 conjugated to phosphatidylethanolamine (PE) is bound to the phagophore membrane and it interacts with several ATG proteins and also with cargo receptor proteins, such as NBR1 [69,85,86]. Together with the cargoes, a fraction of ATG8-PE is delivered to the vacuole by the matured autophagosome for degradation.

We have previously found that ATG11 selective autophagy scaffold protein is recruited by the TBSV p33 replication protein into VROs [87]. The co-opted ATG11 facilitates the formation of vMCS, thus playing a pro-viral role. The findings that tombusviruses usurp a key selective autophagy protein for pro-viral functions open the question: Do tombusviruses usurp additional autophagy proteins or membranes?

In this work, we demonstrate that the key autophagy protein, ATG8, is recruited into TBSV VROs via interaction with the p33 replication protein. Moreover, NBR1 selective autophagy protein is also subverted by p33. Knockdown of ATG8f or NBR1 in *Nicotiana benthamiana* led to decreased TBSV and CIRV replication, suggesting pro-viral function of selective autophagy in tombusvirus replication. Co-opting the above autophagy proteins into the VROs resulted in reduced autophagic flux, suggesting that tombusviruses regulate the autophagy pathway in plants. We observed that a fraction of the usurped ATG8f or NBR1 formed biomolecular condensates associated with VROs, likely trapping those proteins away from the general autophagy pathway. Overall, tombusviruses hijack selective autophagy to provide phospholipid-rich membranes for replication and to regulate the antiviral authophagic flux.

## Results

### Recruitment of ATG8 into tombusvirus VROs in plants

**Fig 1:**
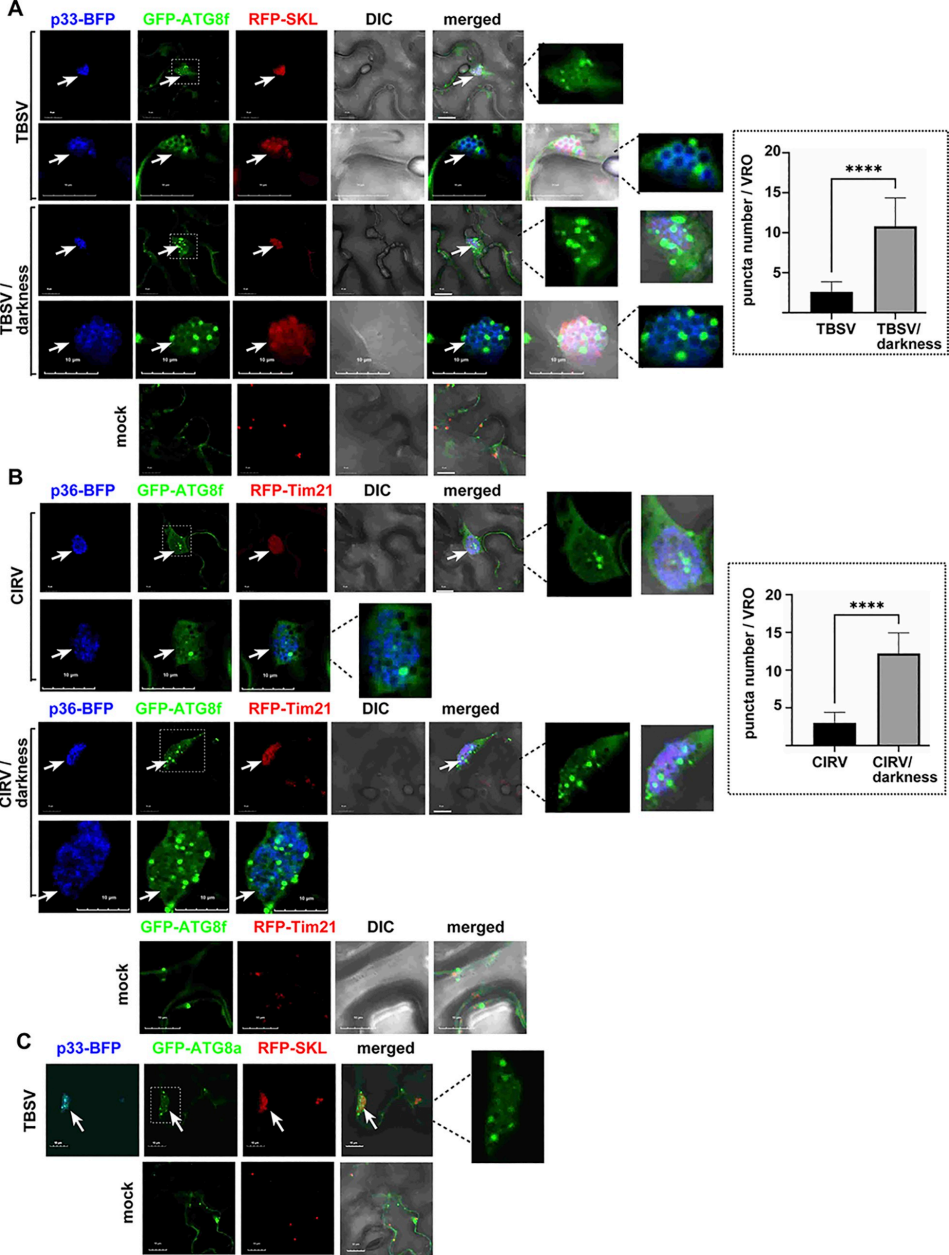
Recruitment of ATG8 by the TBSV p33 and the CIRV p36 replication proteins into VROs in *N*. *benthamiana*. (A) Confocal microscopy images show efficient co-localization of TBSV p33-BFP replication protein and GFP-ATG8f within VROs consisting of clustered peroxisomes, marked by RFP-SKL peroxisomal matrix marker in *N*. *benthamiana* leaves. The expression of these proteins, driven by the 35S promoter, was achieved through co-agroinfiltration into *N*. *benthamiana* leaves. The plant leaves were either TBSV-infected and applied darkness treatment (to induce bulk autophagy) or mock-inoculated as shown. The VROs are marked with arrows. The enlarged images of VROs (boxed) are shown on the right. Scale bars represent 10 μm. The graph shows the number of ATG8f puncta / VRO. Error bars represent SD (n  =  10). Student t-test was used to statistically analyze the data (****P < 0.0001). (B) Confocal microscopy images show efficient co-localization of CIRV p36-BFP replication protein and GFP-ATG8f within VROs consisting of clustered mitochondria, marked by RFP-AtTim21 mitochondrial marker in *N*. *benthamiana* leaves. See further details in panel A. (C) Confocal microscopy images show co-localization of TBSV p33-BFP replication protein and GFP-ATG8a within VROs in *N*. *benthamiana* leaves. Note that ATG8a localized in both the cytosol and nucleus in the absence of tombusvirus infection. See further details in panel A. Each experiment was repeated.

To learn if additional autophagy component(s) is usurped by TBSV into VROs, we selected small ubiquitin-like ATG8 key autophagy protein, based on the ability of ATG8 to bind most cargo receptors and core autophagy proteins [83]. Confocal imaging showed that co-expression of GFP-tagged ATG8f2 (called ATG8f hereafter) with BFP-tagged TBSV p33 replication protein and RFP-SKL peroxisomal marker protein in *N*. *benthamiana* cells infected with TBSV led to the accumulation of GFP-ATG8f in TBSV VROs. Higher magnification of the images showed that GFP-ATG8f partially co-localized with the TBSV p33 replication protein in the VRO representing clustered peroxisomes (Fig 1A, top panels). GFP-ATG8f also formed small punctate structures within the VROs. Induction of autophagy via darkness treatment of *N*. *benthamiana* plants [88] did not interfere with the recruitment of GFP-ATG8f into VRO (Fig 1A, middle panels). However, we noted ~4-fold increased number of small punctate structures containing GFP-ATG8f within the VROs after darkness treatment. GFP-ATG8f was localized in the cytosol of *N*. *benthamiana* cells in the absence of TBSV infection (Fig 1A).

Confocal microscopy-based experiments showed that the closely-related CIRV, which usurps clustered mitochondria for replication, co-opted GFP-ATG8f into VROs marked with the CIRV p36-BFP replication protein and RFP-Tim21 mitochondrial membrane protein (Fig 1B, top panels). After darkness treatment of *N*. *benthamiana*, the localization pattern of GFP-ATG8f within the CIRV VROs was similar to the pattern observed with TBSV VROs (compare Fig 1A and 1B). Also, we counted ~4-fold increased number of small punctate structures containing GFP-ATG8f within the CIRV VROs after darkness treatment.

To test if additional members of the ATG8 protein family were also co-opted by TBSV, we selected ATG8a and ATG8i. Interestingly, both GFP-ATG8a and GFP-ATG8i were usurped into TBSV and CIRV VROs, forming small number of punctate structures (Figs 1C and S1A–S1C). We also found that the minus stranded replicon RNA, which is the replication intermediate and marks the site of virus replication [49,89] was co-localized with p33 replication protein and ATG8a in the replication compartment in *N*. *benthamiana* replicating the closely-related cucumber necrosis (CNV) helper virus, which supplied the RdRp (S2 Fig). Therefore, we suggest that ATG8a is recruited to VROs that are active in viral RNA synthesis. Based on these experiments, we conclude that ATG8 autophagy-related proteins are efficiently recruited by tombusviruses to VROs in *N*. *benthamiana*.

To test if additional core autophagy proteins were recruited into tombusvirus VROs, we performed confocal microscopy experiments, which revealed that GFP-ATG4, GFP-ATG1a, GFP-ATG101, and GFP-ATG5 core autophagy proteins were co-localized with TBSV p33 replication proteins within VROs marked by RFP-SKL peroxisomal marker (S3 Fig). We also tested the plant specific autophagy adaptor protein SH3P2, a BAR domain protein, which participates in membrane deformation of phagophore assembly site [90,91]. Moreover, SH3P2 is targeted by the *Xanthomonas* effector protein, XopL, for degradation to suppress autophagy [92]. Interestingly, GFP-SH3P2 is re-localized to the VROs in the presence of p33 replication protein (S3 Fig). Taken together, several autophagy proteins are re-localized to TBSV VROs, suggesting that TBSV hijacks the autophagy machinery in plant cells.

### Tombusvirus replication proteins interact with ATG8 in plants

**Fig 2:**
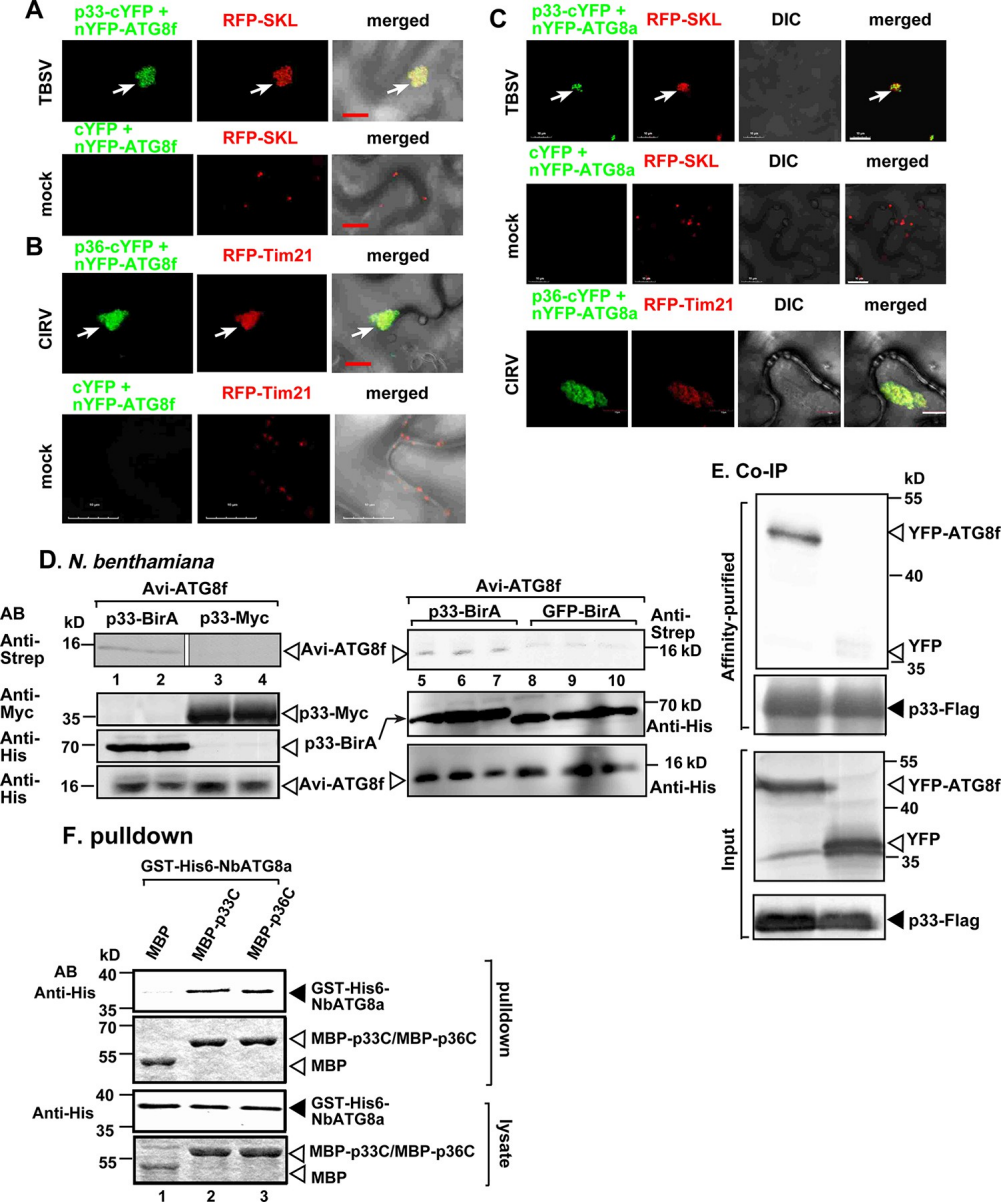
Interaction between ATG8 and tombusvirus replication proteins within VROs in *N*. *benthamiana*. (A) Interaction between TBSV p33-cYFP replication protein and the nYFP-ATG8f protein was detected by BiFC. The merged images show the co-localization of RFP-SKL with the BiFC signal, indicating that the interaction between p33 replication protein and ATG8f occurs in VROs in clustered peroxisomal membranes. The VROs are marked with arrows. Scale bars represent 10 μm. Each experiment was repeated three times. (B) Interaction between CIRV p36-cYFP replication protein and the nYFP-ATG8f protein were detected by BiFC. The merged images show the co-localization of RFP-AtTim21 with the BiFC signal, indicating that the interaction between p36 replication protein and ATG8f occurs in VROs consisting of aggregated mitochondria. See further details in panel A. (C) Interactions between TBSV p33-cYFP or CIRV p36-cYFP replication proteins and the nYFP-ATG8a protein were detected by BiFC. See further details in panel A. (D) Protein proximity-labeling with biotin in *N*. *benthamiana*. *N*. *benthamiana* leaves were agroinfiltrated to express p33 replication protein, which was fused to BirA biotin ligase, and Avi-tagged ATG8f. Note that both fusion proteins carry a 6xHis tag. Biotin treatment lasted for 40 min. The image shows the western blot analysis of the biotinylated Avi-ATG8f detected with streptavidin-conjugated AP in total protein extracts. p33-myc was used as a negative control to show plant does not have biotin ligase activity. GFP-BirA was expressed (lanes 8–10) to measure basal level of biotinylation of Avi-ATG8f. The experiments were repeated. (E) Co-purification of YFP-ATG8f with TBSV Flag-p33 replication protein from subcellular membranes. *N*. *benthamiana* leaves were agroinfiltrated to express YFP-ATG8f with TBSV Flag-p33. Top two panels: western blot analysis of co-purified YFP-ATG8f detected with anti-YFP antibody, while Flag-p33 was detected with anti-Flag antibody. The negative control was from plants expressing YFP purified on a Flag-affinity column. Bottom two panels: western blot of input YFP-ATG8f, YFP and Flag-p33 in the total protein extracts. (F) Pull-down assay including His_6_-NbATG8a and the MBP-tagged TBSV p33 or CIRV p36 replication proteins. Note that we used the soluble C-terminal region of TBSV p33 or CIRV p36 replication proteins, which lacked the N-terminal domain. Top panel: western blot analysis of the captured His_6_-NbATG8a with the MBP-affinity purified p33C/p36C was performed with anti-His antibody. The negative control was the MBP (lanes 1). Middle panel: Coomassie-blue stained SDS-PAGE of the captured MBP-p33C, MBP-p36C and MBP. Bottom panels: western blot analysis of His_6_-NbATG8a in total extracts. Coomassie-blue stained SDS-PAGE of the MBP-p33C, MBP-p36C and MBP in total extracts. Each experiment was repeated three times.

To test if the TBSV p33 replication protein interacts with ATG8f, bimolecular fluorescence complementation (BiFC) experiments were conducted in *N*. *benthamiana* leaves. The BiFC signals revealed specific interaction between TBSV p33 replication protein and ATG8f and the interaction occurred in the VROs marked with RFP-SKL peroxisomal marker (Fig 2A, and the bottom panel for the negative control experiment). BiFC assays showed that the CIRV p36 replication protein interacted with ATG8f within the VROs (Fig 2B). Similar experiments also showed interactions between the TBSV p33 or CIRV p36 replication proteins and ATG8a (Fig 2C), and TBSV p33 or CIRV p36 and ATG8i (S1D Fig) in VROs. Thus, bulk of the interactions between the tombusviral replication proteins and ATG8 takes place within the VROs in *N*. *benthamiana*.

To confirm interactions between the TBSV p33 replication protein and ATG8f, we performed protein proximity-labeling approach. This was based on *E*. *coli*-derived BirA biotin-ligase and Avi tag, which serves as a biotin acceptor peptide [93,94]. The BirA was fused to p33, which targets the fusion protein to VROs [87]. The Avi tag was fused to ATG8f to monitor proximity to p33-BirA in plant cells. Co-expression of p33-BirA and Avi-ATG8f in *N*. *benthamiana* led to biotinylation of Avi-ATG8f (Fig 2D, lanes 1–2). Co-expression of Myc-tagged p33 (absence of the BirA fusion), and Avi-ATG8f did not lead to its biotinylation in *N*. *benthamiana*, thus excluding endogenous biotin ligase activity in plant (Fig 2D, lanes 3–4). GFP-BirA was expressed in *N*. *benthamiana* to measure basal level of biotinylation of Avi-ATG8f (Fig 2D lanes 8–10). Altogether, the above data confirm the close proximity of ATG8f autophagy protein to the tombusvirus p33 replication protein in plant cells.

To further confirm interactions between the TBSV p33 replication protein and ATG8, we performed three additional assays. The first one was based on affinity-purification of Flag-p33 from detergent-solubilized membrane fraction of plants expressing YFP-ATG8f (Fig 2E) or His_6_-ATG8a (S4A Fig) [49]. The negative control was plants expressing His_6_-p33 and YFP or His_6_-ATG8a. The second interaction assay was a pull-down assay with MBP-tagged p33 or MBP-p36 and GST-6xHis-tagged ATG8a purified from *E*. *coli* (Fig 2F). For the pulldown assay, we used N-terminally truncated TBSV p33 and CIRV p36 replication proteins lacking their membrane-binding regions to aid their solubility in *E*. *coli*. Altogether, the pulldown data suggest that the replication proteins of TBSV and CIRV use their C-terminal domains facing the cytosolic compartment to directly interact with ATG8a protein *in vitro*. Additional pulldown experiments also showed that the very N-terminal region of TBSV p33 (1–82 aa, lacking the membrane-binding regions) bound with ATG8f (S4B Fig). Mutagenesis of the predicted ATG8-binding motif (AIM1) within the N-terminal region did not completely eliminate binding to ATG8f, whereas deletion of the N-terminal 36 aa eliminated binding to ATG8f (S4B Fig). These data suggest that p33 sequences outside of AIM1 contribute to binding to ATG8f. The third interaction assay was based on the split-ubiquitin-based membrane yeast two-hybrid assay (MYTH) [95]. We found that both ATG8a protein and the yeast ScATG8 protein interacted with the full-length TBSV p33 replication protein in yeast (S4C Fig).

Protein proximity-labeling also showed close proximity of ATG4, ATG101 and ATG5 autophagy proteins to the tombusvirus p33 replication protein in plant cells (S5A Fig). We confirmed the interactions of ATG4, ATG5 and ATG101 with the TBSV p33 replication protein using affinity-purification of Flag-p33 from detergent-solubilized membrane fraction of plants (S5B Fig). BiFC studies demonstrated interactions between TBSV p33 and SH3P2 autophagy adaptor protein and ATG8f and SH3P2 in the VROs (S6A and S6B Fig). These data suggest that the TBSV p33 replication protein actively recruits several autophagy proteins via protein-protein interactions into VROs.

### ATG8 autophagy protein facilitates tombusvirus replication in plants

**Fig 3:**
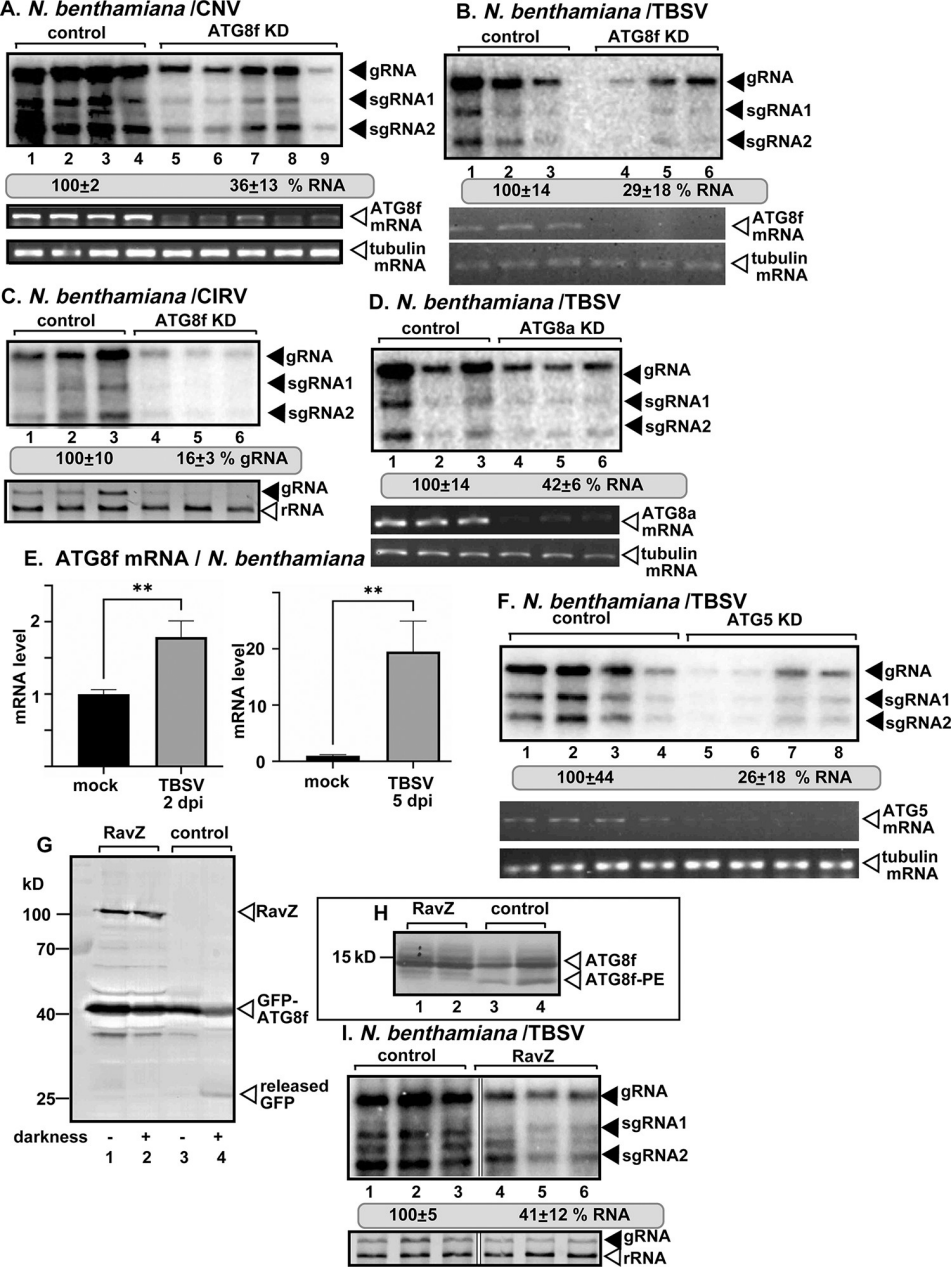
The effect of ATG8 on tombusvirus replication in *N*. *benthamiana* plants. (A-B) Top panel: The accumulation of the CNV and TBSV RNAs in ATG8f-silenced (ATG8f KD) *N*. *benthamiana* plants 2.5 and 2 dpi, respectively. The genomic (g)RNA levels in the inoculated leaves were measured by northern blot analysis. Agroinfiltration of pGD-CNV^20Kstop^ or inoculation with TBSV sap was done 10 days after silencing of ATG8f expression. Agroinfiltration of tobacco rattle virus (TRV) vectors carrying either ATG8f or 3′-terminal GFP (as a control) sequences was used to induce VIGS. Second panel: RT-PCR analysis of ATG8f mRNA level in the silenced and control plants. Third panel: RT-PCR analysis of tubulin mRNA level in the silenced and control plants. (C) Top panel: The accumulation of the CIRV RNAs in ATG8f-silenced (ATG8f KD) *N*. *benthamiana* leaves at 3 dpi. See more details in panel A. Bottom panel: Ribosomal (r)RNA is shown as a loading control in an ethidium-bromide-stained agarose gel. (D) The accumulation of the TBSV RNAs in ATG8a-silenced (ATG8a KD) *N*. *benthamiana* leaves at 2 dpi. See more details in panel A. (E) Real time RT-qPCR analysis of the expression of ATG8f mRNA in the TBSV-inoculated leaves (2 dpi) or systemically infected leaves (5 dpi) of *N*. *benthamiana* plants. The same amounts of total RNA extracts were used in RT-qPCR analysis. (F) Accumulation of the TBSV RNAs in ATG5-silenced (ATG5 KD) *N*. *benthamiana* leaves at 2 dpi. See more details in panel A. (G) The effect of *Legionella* RavZ effector on autophagy flux. The plants expressed GFP-ATG8f and either exposed to darkness to induce bulk autophagy or not as shown. The released ‘free’ GFP band is marked with an arrowhead. The left two lanes include samples from plants co-expressing RavZ and GFP-ATG8f, which show lack of released ‘free’ GFP band. Total protein extracts were immunoblotted with anti-GFP antibody. The RavZ and GFP-ATG8f proteins were expressed via agroinfiltration. (H) The effect of *Legionella* RavZ effector on ATG8f-PE conjugation. The two lanes on the right display plants expressing Flag-ATG8f, where both Flag-ATG8f and Flag-ATG8f-PE are present as marked by arrowhead. The left two lanes show that RavZ expression eliminated the Flag-ATG8f-PE band, but not the Flag-ATG8f band. Total protein extracts were loaded on 15% polyacrylamide gels containing 6 M urea and immunoblotted with anti-Flag antibody. (I) Top panel: The accumulation of the TBSV RNAs in *N*. *benthamiana* leaves expressing RavZ at 2 dpi. The RavZ protein was expressed via agroinfiltration. The control leaves were agroinfiltrated with the same amounts of bacteria with ‘empty’ plasmid. See more details in panel A. Bottom panel: Ribosomal RNA is shown as a loading control in an ethidium-bromide-stained agarose gel. Each experiment was repeated three times.

The robust recruitment of ATG8 autophagy protein into VROs might have an effect on tombusvirus replication. To test this possibility, we silenced ATG8f expression using virus-induced gene-silencing (VIGS) in *N*. *benthamiana* plants. The region selected in NbATG8f was specific to the highly similar f1 and f2 members of the ATG8 gene family. Knockdown of ATG8f in *N*. *benthamiana* led to ~3-fold reduction of TBSV and the closely related cucumber necrosis virus (CNV^20KStop^), which does not express the gene silencing suppressor protein replication, and ~6-fold reduction of CIRV RNA accumulation in the inoculated leaves (Fig 3A–3C). Knockdown of ATG8f level did not cause obvious phenotype in *N*. *benthamiana*. Silencing of ATG8f expression did not affect the expression of ATG8a or ATG8i (S7 Fig). Knockdown of ATG8a in *N*. *benthamiana* plants also reduced TBSV RNA accumulation by >2-fold (Fig 3D), whereas knockdown of ATG8i did not have much effect on TBSV replication (S1E Fig). Based on the most pronounced effect of ATG8f knockdown on tombusvirus replication, we focused on ATG8f in subsequent studies.

To test if ATG8f expression is affected during TBSV infection in *N*. *benthamiana* plants, RT-qPCR analysis was performed. Comparison of ATG8f mRNA levels in TBSV-infected versus mock-treated *N*. *benthamiana* leaves revealed 2-fold up-regulation of ATG8f mRNA level in the TBSV inoculated leaves 2 days after inoculation (dpi) (Fig 3E). Interestingly, ATG8f mRNA level was ~20-fold higher in systemically-infected leaves in comparison with similar leaves in the control uninfected plants (Fig 3E). These data indicate that TBSV replication induces high level expression of ATG8f in *N*. *benthamiana* plants.

During autophagy process, ATG8 becomes lipidated by the ATG12-ATG5-ATG16 complex, making ATG8-PE (phosphatidylethanolamine), which is membrane bound and shows enhanced activities [83]. To test if ATG8 lipidation is important during TBSV infections, we knocked down ATG5 via VIGS in *N*. *benthamiana*. TBSV RNA accumulation was reduced by ~4-fold in ATG5 knockdown plants (Fig 3F), suggesting a role of ATG8 lipidation in TBSV replication. To test the combined role of the ATG8 family members and ATG8 lipidation in TBSV replication, we expressed RavZ protease, which is an effector protein from *Legionella* bacterium [96,97]. RavZ has been shown to cleave lipidated LC3 (an ATG8 ortholog) of the mammalian host, effectively destroying LC3/ATG8 function in autophagy [96,97]. We confirmed that expression of RavZ protease inhibited general autophagy in *N*. *benthamiana* based on ‘free’ GFP analysis using GFP-ATG8f (Fig 3G, lane 2 versus 4). This assay is based on that autophagy pathway sends GFP-ATG8f into the vacuole for degradation. However, the GFP portion of the fusion protein is relatively stable in the vacuole and the protease-driven degradation process results in the release of ‘free’ GFP from the fusion protein, which can be detected via western blotting [98]. In addition, expression of RavZ eliminated the ATG8f-PE form in plant cells (Fig 3H, lanes 1–2 versus 3–4). We found that expression of RavZ inhibited TBSV replication by >2-fold in *N*. *benthamiana* (Fig 3I). Altogether, the above data confirmed that ATG8 members, especially ATG8f, play pro-viral roles in tombusvirus replication in plants. Moreover, the inhibitory effect of RavZ effector expression on TBSV replication also supported the role of lipidated ATG8.

### NBR1 selective autophagy receptor is recruited into TBSV VROs

**Fig 4:**
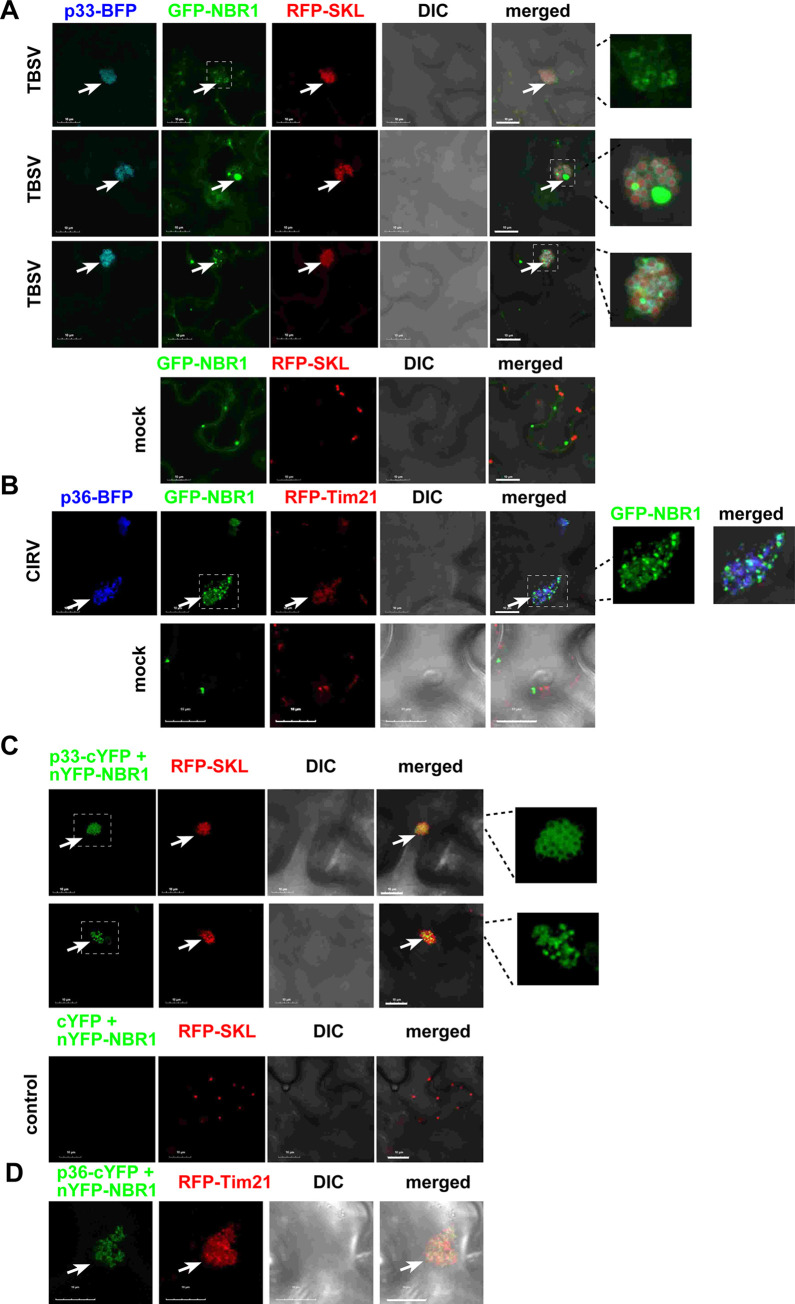
Recruitment of NBR1 selective autophagy receptor by the TBSV p33 and the CIRV p36 replication proteins into VROs in *N*. *benthamiana*. (A) Confocal microscopy images show co-localization of TBSV p33-BFP replication protein and the eGFP-NBR1 within a VRO consisting of clustered peroxisomes, marked by RFP-SKL peroxisomal matrix marker in *N*. *benthamiana* leaves. The expression of these proteins, driven by the 35S promoter, was achieved through co-agroinfiltration into *N*. *benthamiana* leaves. The plant leaves were either TBSV-infected, or mock-inoculated as shown. Note that eGFP-NBR1 formed different patterns as visible in the enlarged images on the right. Scale bars represent 10 μm. See further details in Fig 1A. (B) Confocal microscopy images show co-localization of CIRV p36-BFP replication protein and the eGFP-NBR1 within a VRO consisting of clustered mitochondria, marked by RFP-AtTim21 mitochondrial marker in *N*. *benthamiana* leaves. See further details in panel A. (C) Interaction between TBSV p33-cYFP replication protein and the nYFP-NBR1 protein was detected by BiFC. The merged images show the co-localization of RFP-SKL with the BiFC signals, indicating that the interaction between p33 replication protein and NBR1 occurs in VROs. The interacting proteins formed different patterns as visible in the enlarged images on the right. (D) Interaction between CIRV p36-cYFP replication protein and the nYFP-NBR1 protein was detected by BiFC. See further details in panel C above. Each experiment was repeated.

Because ATG8 participates in both bulk and selective autophagy pathways [83], we tested if TBSV usurps NBR1 selective autophagy receptor [69,86,99] into VROs. Confocal imaging showed that co-expression of eGFP-NBR1 with BFP-tagged TBSV p33 replication protein and RFP-SKL peroxisomal marker protein in *N*. *benthamiana* cells infected with TBSV led to the accumulation of eGFP-NBR1 in TBSV VROs (Fig 4A). Interestingly, eGFP-NBR1 formed different patterns within the VROs. These included small punctate structures within the TBSV and CIRV VROs (Fig 4A and 4B), similar to those seen with ATG8f (Fig 1A). Several puncta showed the co-localization of eGFP-NBR1 with the replication proteins (see enlarged panel Fig 4A and 4B), whereas other puncta were enriched with mostly eGFP-NBR1. eGFP-NBR1 also formed larger puncta, which did not co-localize with p33 replication protein, but were associated with the VROs (Fig 4A, central panel). BiFC experiments showed interaction between TBSV p33 and NBR1 within the VROs (Fig 4C). Two types of patterns were present in BiFC images, one forming mostly circles around the peroxisomes (top image, Fig 4C) and the other type showing small puncta (bottom image, Fig 4C). To confirm interactions between the TBSV p33 replication protein and NBR1, we performed protein proximity-labeling approach with p33-BirA and Avi-NBR1. Co-expression of p33-BirA and Avi-NBR1 in *N*. *benthamiana* led to biotinylation of Avi-NBR1 (S5 Fig, lanes 13–14). Co-expression of Myc-tagged p33 (absence of the BirA fusion), and Avi-NBR1 did not lead to its biotinylation in *N*. *benthamiana* (S5 Fig, lanes 15–16). Altogether, the above data confirm the close proximity of NBR1 selective autophagy receptor to the tombusvirus p33 replication protein in plant cells. Interaction between CIRV p36 replication protein and NBR1 was also observed by BiFC (Fig 4D). These data support the model that TBSV and CIRV hijack NBR1, and the selective autophagy.

**Fig 5:**
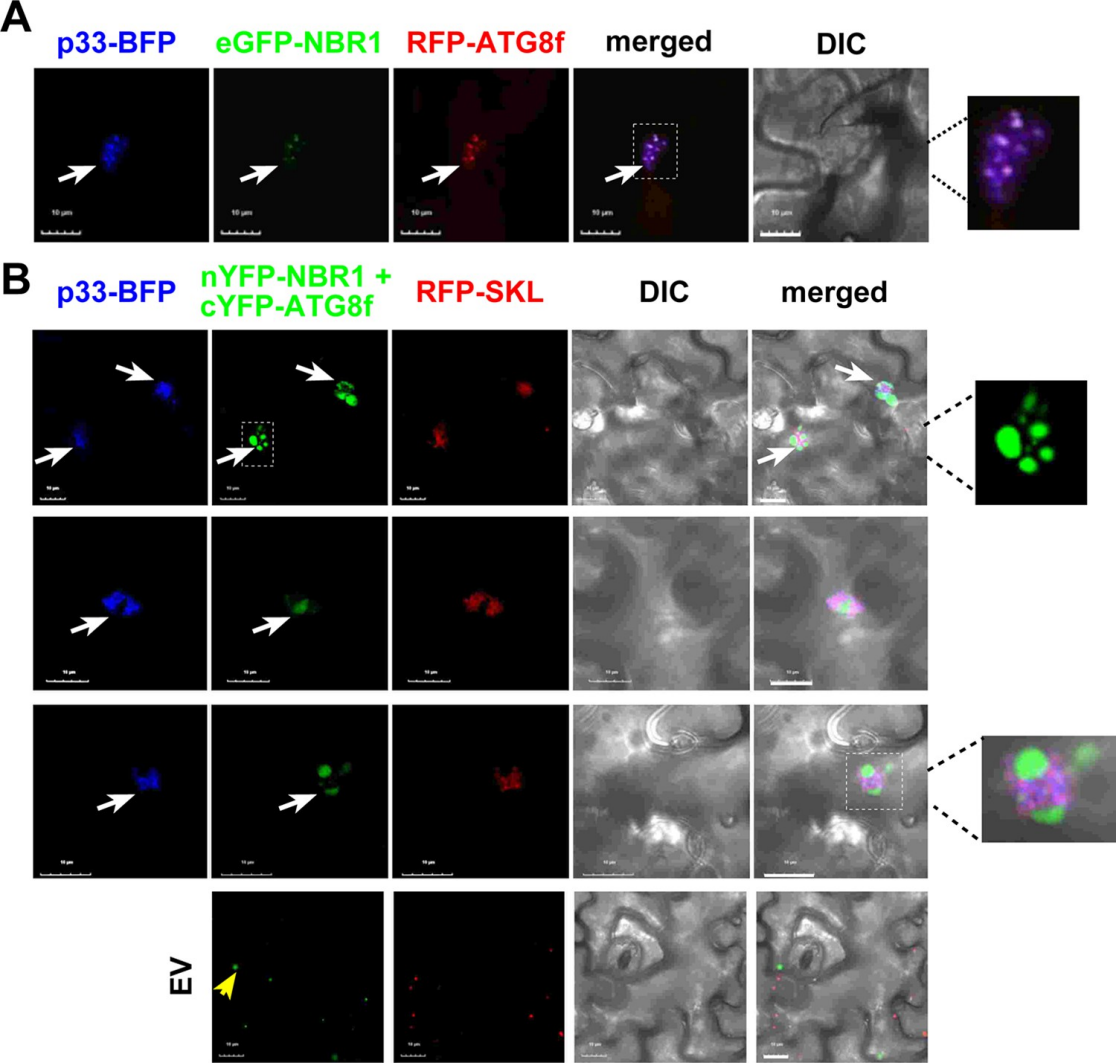
Interaction between the co-opted ATG8f and NBR1 proteins within VROs in *N*. *benthamiana*. (A) Confocal microscopy images show co-localization of TBSV p33-BFP replication protein and the co-opted RFP-ATG8f and eGFP-NBR1 within a VRO consisting of clustered peroxisomes in *N*. *benthamiana* leaves. The expression of these proteins, driven by the 35S promoter, was achieved through co-agroinfiltration into *N*. *benthamiana* leaves. The VROs are marked with arrows. Scale bars represent 10 μm. (B) BiFC approach was employed to demonstrate interaction between nYFP-NBR1 and cYFP-ATG8f proteins associated with the TBSV p33-BFP-positive VROs. Co-agroinfiltration into *N*. *benthamiana* leaves was used for protein expression. ‘EV’ represents plant cells not expressing p33-BFP. Scale bars represent 10 μm. Each experiment was repeated.

NBR1 is known to interact with ATG8f in plant cells [69,99]. Indeed, we observed that eGFP-NBR1 co-localized with RFP-ATG8f and p33-BFP replication protein in small puncta within the VROs (Fig 5A). Using BiFC by co-expressing nYFP-NBR1 and cYFP-ATG8f in *N*. *benthamiana* infected with TBSV, we found that the co-opted NBR1 and ATG8f interacted within the VROs marked with TBSV p33-BFP (Fig 5B). Interestingly, the interacting eGFP-NBR1 and RFP-ATG8f were mostly present in punctate structures associated with the VROs. Moreover, when eGFP-NBR1 and RFP-ATG8f together formed larger puncta, then p33 replication protein and the co-opted peroxisomes seemed to be excluded from the puncta. However, the puncta and the co-opted peroxisomes were always located in close vicinity within the VROs (Fig 5B). This suggests maturation of eGFP-NBR1 and RFP-ATG8f puncta into “bodies” sequestering eGFP-NBR1 and RFP-ATG8f within VROs.

**Fig 6:**
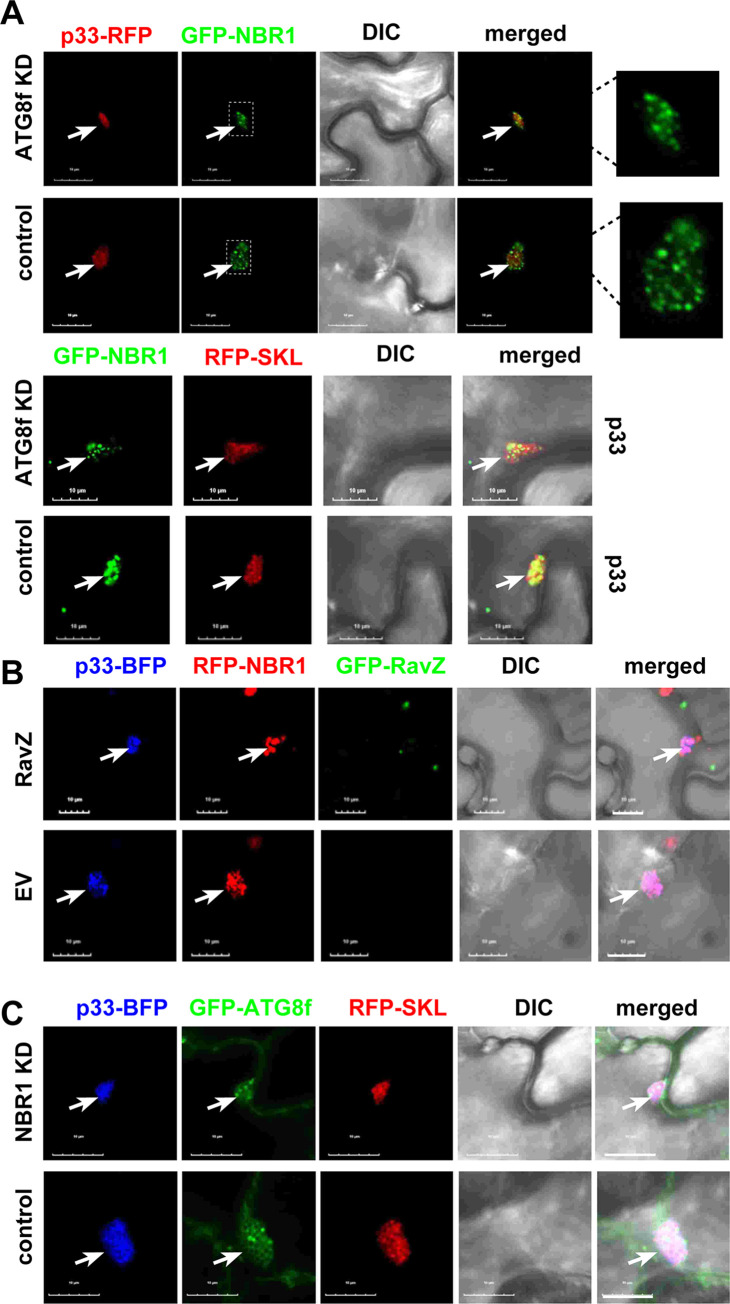
Separate subversion of ATG8f or NBR1 by TBSV p33 replication protein into VROs. (A) Confocal microscopy images show co-localization of eGFP-NBR1 and p33-RFP (top two panels), or eGFP-NBR1 and RFP-SKL co-expressing cYFP-p33 (bottom panels), within VROs in ATG8f-silenced (ATG8f KD) or control *N*. *benthamiana* cells. The expression of these proteins, driven by the 35S promoter, was achieved through co-agroinfiltration into *N*. *benthamiana* leaves. TRV vector carrying MBP sequences was used as a VIGS control. Scale bars represent 10 μm. The VROs are marked with arrows. (B) Confocal microscopy images show co-localization of RFP-NBR1 and p33-BFP in *N*. *benthamiana* cells. The plants either expressed GFP-RavZ effector (top panel), or pGD vector as control (bottom panel). See further details in panel A. (C) Confocal microscopy images show co-localization of GFP-ATG8f and p33-BFP in NBR1-silenced (NBR1 KD) *N*. *benthamiana* cells. See more detail in panel A. Scale bars represent 10 μm. Each experiment was repeated.

To test if ATG8f affects the recruitment of NBR1 into the VROs, we knocked down ATG8f levels via VIGS in *N*. *benthamiana* infected with TBSV. Confocal microscopy analysis showed that eGFP-NBR1 was efficiently recruited into the VROs marked either by p33-BFP or RFP-SKL in ATG8f knockdown cells (Fig 6A). Expression of RavZ protease, which eliminates ATG8-PE (Fig 3H), did not interfere with the subversion of NBR1 to the TBSV VROs (Fig 6B). Knocking down NBR1 levels via VIGS in *N*. *benthamiana* infected with TBSV did not seem to inhibit the recruitment of GFP-ATG8f into VROs (Fig 6C). Expression of RavZ protease did not interfere with the subversion of ATG8f to the TBSV VROs (S8 Fig), suggesting that TBSV p33 could recruit the nonlipidated ATG8f to the VROs. Thus, NBR1 and ATG8f are co-opted by p33 replication protein seemingly separately into VROs. However, we cannot exclude that the co-opted NBR1 and ATG8f is recruited as a complex by p33 into VROs in WT plants.

### The co-opted NBR1 autophagy receptor promotes tombusvirus replication in plants

**Fig 7:**
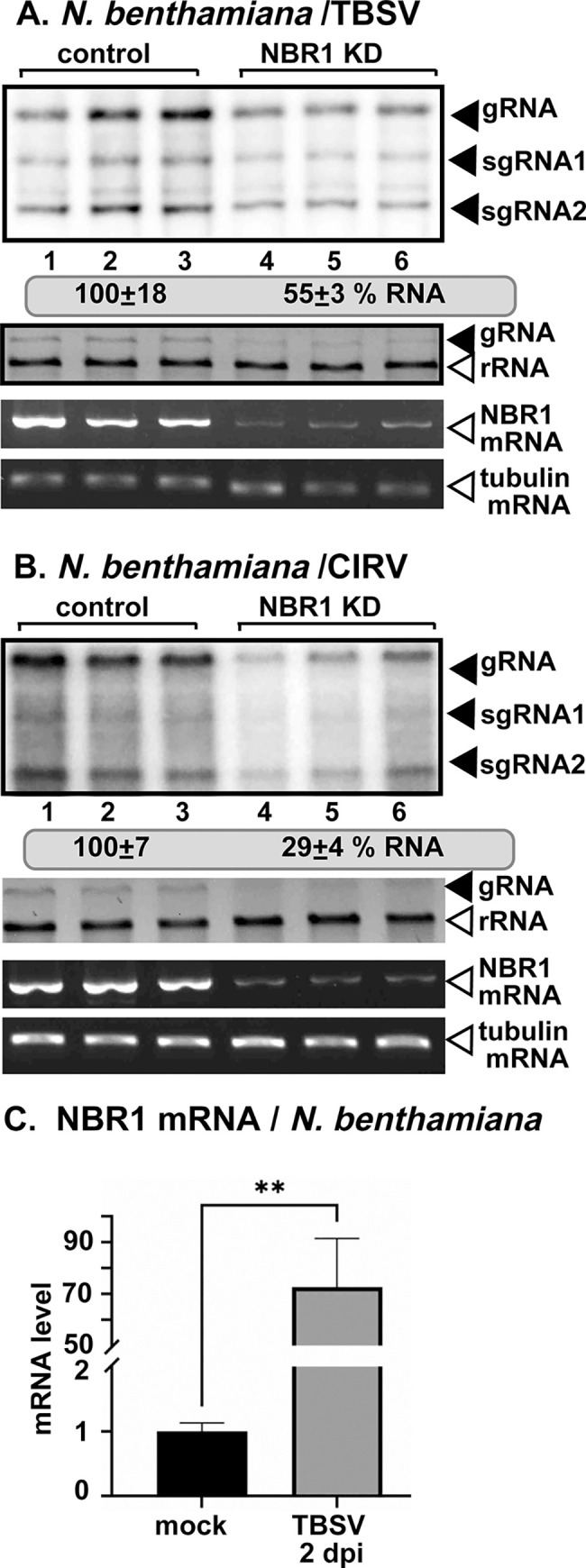
The effect of NBR1 on tombusvirus replication in *N*. *benthamiana* plants. (A) Top panel: The accumulation of TBSV RNAs in NBR1-silenced (NBR1 KD) inoculated leaves of *N*. *benthamiana* at 2 dpi was measured by northern blot analysis. Sap inoculation with TBSV was done 10 days after silencing of NBR1 expression. Agroinfiltration of TRV vector carrying NBR1 or 3’-terminal GFP (as a control) sequences was used to induce VIGS. Second panel: Ribosomal RNA is shown as a loading control in an ethidium-bromide stained agarose gel. Third panel: RT-PCR analysis of NBR1 mRNA level in the silenced and control plants. Fourth panel: RT-PCR analysis of tubulin mRNA level in the silenced and control plants. Each experiment was repeated three times. (B) The accumulation of CIRV RNAs in NBR1-silenced (NBR1 KD) inoculated leaves of *N*. *benthamiana* at 2.5 dpi was measured by northern blot analysis. Sap inoculation with CIRV was done 10 days after silencing of NBR1 expression. See further details in panel A above. (C) Real time RT-qPCR analysis of the induction of NBR1 mRNA expression in the TBSV-inoculated leaves (2 dpi) of *N*. *benthamiana* plants. The same amounts of total RNA extracts were used in RT-qPCR analysis. Each experiment was repeated three times.

Knockdown of NBR1 in *N*. *benthamiana* via VIGS resulted in ~2-fold decrease in TBSV replication and >3-fold reduction of CIRV RNA accumulation in the inoculated leaves (Fig 7A and 7B). Knockdown of NBR1 mRNA level did not cause phenotype in *N*. *benthamiana*. These experiments suggest that subversion of NBR1 by p33 has a pro-viral role in tombusvirus replication in plants.

To test if TBSV infection affects NBR1 expression in *N*. *benthamiana* plants, we used RT-qPCR analysis, which showed increased production of NBR1 mRNA at 48 h post inoculation in comparison with mock-treated *N*. *benthamiana* leaves (Fig 7C). These data suggest that TBSV replication induces NBR1 expression in *N*. *benthamiana* plants.

### Enrichment of PE in TBSV VRO membrane is affected by ATG8 and NBR1

**Fig 8:**
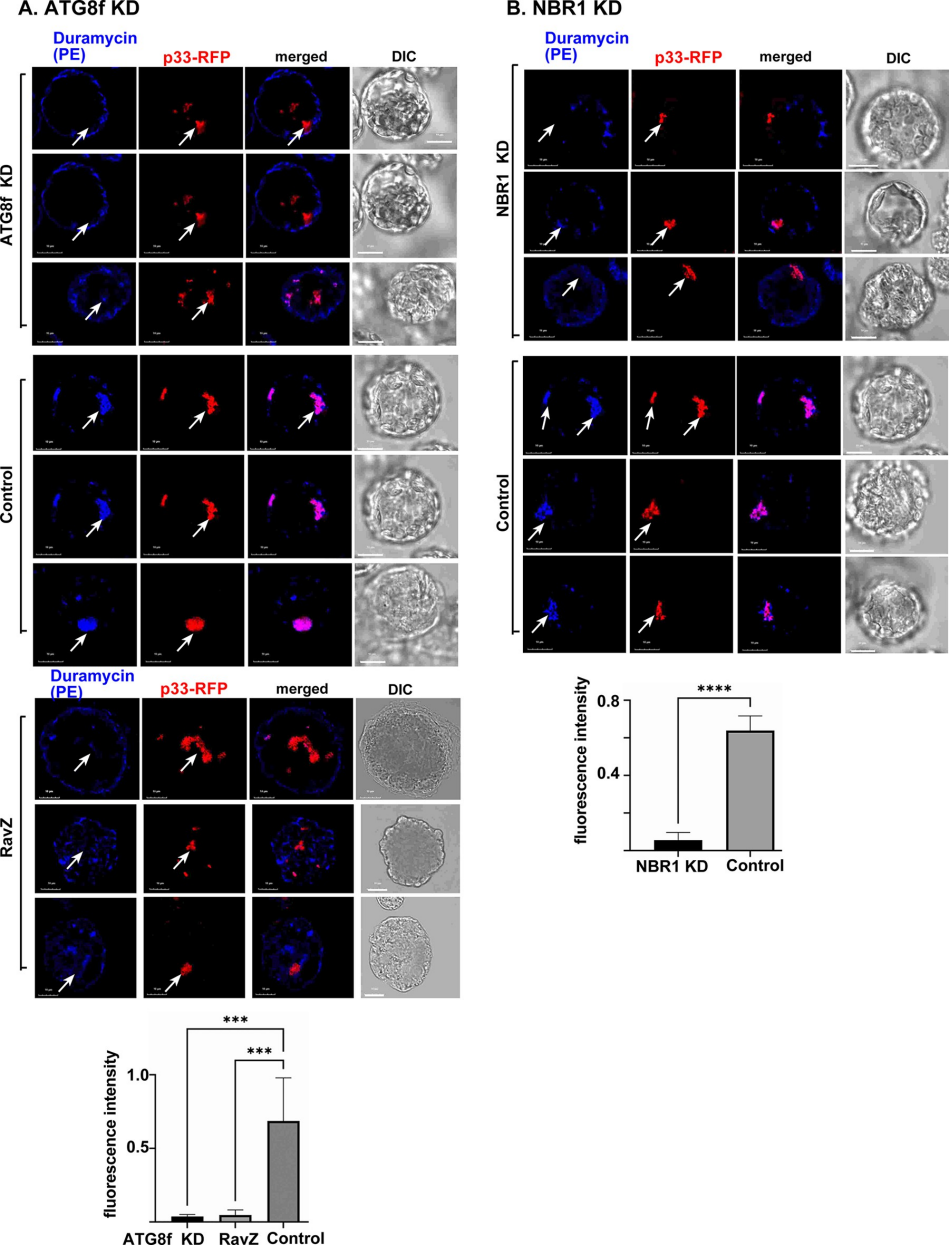
Contributions of ATG8f and NBR1 to PE enrichment in the viral replication compartment in *N*. *benthamiana* protoplasts. (A) Top panel: Confocal microscopy images reveal poor PE enrichment in VROs marked with p33-RFP in protoplasts prepared from ATG8f-silenced (ATG8f-KD) *N*. *benthamiana* (top image). Central panel: PE enrichment is visible in VROs in control *N*. *benthamiana* protoplasts. Differential interference contrast (DIC) images are shown on the right. PE distribution is detected by a staining probe using biotinylated duramycin peptide and streptavidin conjugated with Alexa Fluor 405. Bottom panel: Confocal microscopy images show poor PE re-distribution into VROs in *N*. *benthamiana* protoplasts co-expressing RFP-p33 and RavZ. Scale bars represent 10 μm. The fluorescence intensity of Duramycin was quantified within the VROs marked with arrows using Image J. Error bars represent SD (n  =  10). Student t-test was used for statistical analysis (***P < 0.001, ****P < 0.0001). (B) Confocal microscopy images show poor PE re-distribution into VROs marked with p33-RFP in NBR1-silenced (NBR1-KD) *N*. *benthamiana* protoplasts (top image). TRV vector carrying partial GFP sequences was used as a VIGS control. See more details in panel A above. Each experiment was repeated three times.

We have previously shown that TBSV induces the remarkable enrichment of PE within membranes of VROs [45]. To determine if ATG8 and the selective autophagy pathway plays a role in PE enrichment within VROs, we tested PE distribution in ATG8f and NBR1 knockdown *N*. *benthamiana* protoplasts (single cells without the cell wall) infected with TBSV. Confocal microscopy was used to detect the subcellular distribution of PE by applying biotinylated duramycin peptide and streptavidin conjugated with Alexa Fluor 405 [39,45]. Interestingly, PE enrichment was low within TBSV VROs in ATG8f knockdown protoplasts (Fig 8A), whereas PE was highly enriched within VROs in control protoplasts (Fig 8A). To confirm the role of ATG8 in PE-enrichment in TBSV VROs, we expressed RavZ protease in *N*. *benthamiana* infected with TBSV. Confocal microscopic analysis of protoplasts expressing RavZ protease showed reduced enrichment of PE within the TBSV VROs (Fig 8A). NBR1 knockdown also reduced PE enrichment within TBSV VROs in protoplasts (Fig 8B). Altogether, these data support the role of ATG8f and NBR1 and the selective autophagy pathway in PE enrichment within TBSV VRO membranes in *N*. *benthamiana*. Thus, TBSV utilizes the selective autophagy pathway to contribute PE and membranes to the biogenesis of VROs in plants.

### Enrichment of PI(3)P within the TBSV replication compartment is affected by ATG8 and NBR1

**Fig 9:**
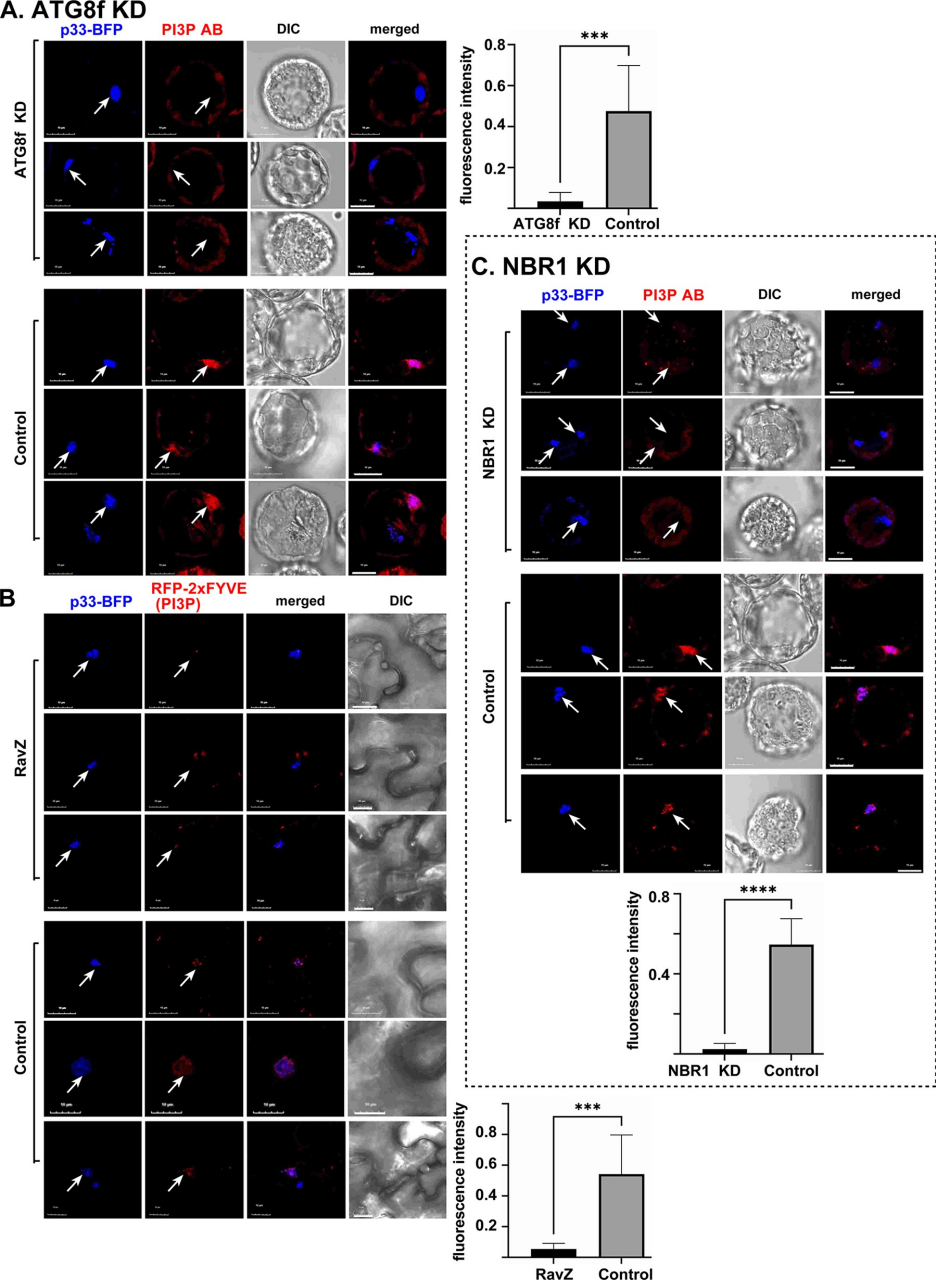
ATG8 and NBR1 contribute to PI(3)P enrichment within the viral replication compartment in *N*. *benthamiana* plants and protoplasts. (A) Confocal microscopy images reveal poor PI(3)P enrichment in VROs marked with p33-BFP in ATG8f-silenced (ATG8f-KD) *N*. *benthamiana* protoplasts. PI(3)P enrichment is visible in VROs in control *N*. *benthamiana* protoplasts. PI(3)P distribution is detected by PI(3)P antibody and then incubated with anti-mouse secondary antibody conjugated with Alexa Fluor 568. Scale bars represent 10 μm. The fluorescence intensity for PI(3)P was quantified within the VROs marked with arrows using Image J. Error bars represent SD (n  =  10). Student t-test was used for statistical analysis (***P < 0.001, ****P < 0.0001). (B) Confocal microscopy images show reduced enrichment of PI(3)P in VROs in *N*. *benthamiana* leaves co-expressing RFP-2xFYVE protein, p33-BFP and RavZ. Note that RFP-2xFYVE selectively binds to PI(3)P. PI(3)P enrichment is visible in VROs in control *N*. *benthamiana* cells. Scale bars represent 10 μm. See more details in panel A above. (C) Confocal microscopy images show PI(3)P distribution in NBR1-silenced (NBR1-KD) or TRV2-cGFP control (bottom image) *N*. *benthamiana* protoplasts expressing p33-BFP. See more details in panel A above.

Previously, we have shown that TBSV infection induces the production and enrichment of PI(3)P within VRO membranes, which facilitates viral replication [44,53,54]. The autophagic membranes are enriched in PI(3)P [83,100]. Therefore, we tested if recruitment of ATG8 or NBR1 by the TBSV p33 replication protein could affect enrichment of VRO membranes with PI(3)P. ATG8f level was knocked down via VIGS and the accumulation of PI(3)P was determined in *N*. *benthamiana* protoplasts infected with TBSV. In comparison with the control protoplasts, PI(3)P accumulation within the TBSV VROs was poor (barely detectable) in ATG8f silenced protoplasts (Fig 9A). We also expressed RavZ protease to destroy ATG8 activities in *N*. *benthamiana* infected with TBSV, followed by detecting PI(3)P accumulation using RFP-2xFYVE biosensor in VROs. RFP-2xFYVE selectively binds to PI(3)P in cells, including plant cells [101,102]. RavZ expression inhibited the accumulation of PI(3)P in TBSV VROs, confirming the role of ATG8 in facilitating PI(3)P production in TBSV VROs (Fig 9B). NBR1 knockdown also reduced PI(3)P accumulation within TBSV VROs in protoplasts (Fig 9C).

### ATG8f promotes the recruitment of VPS34 PI3 kinase into VROs

**Fig 10:**
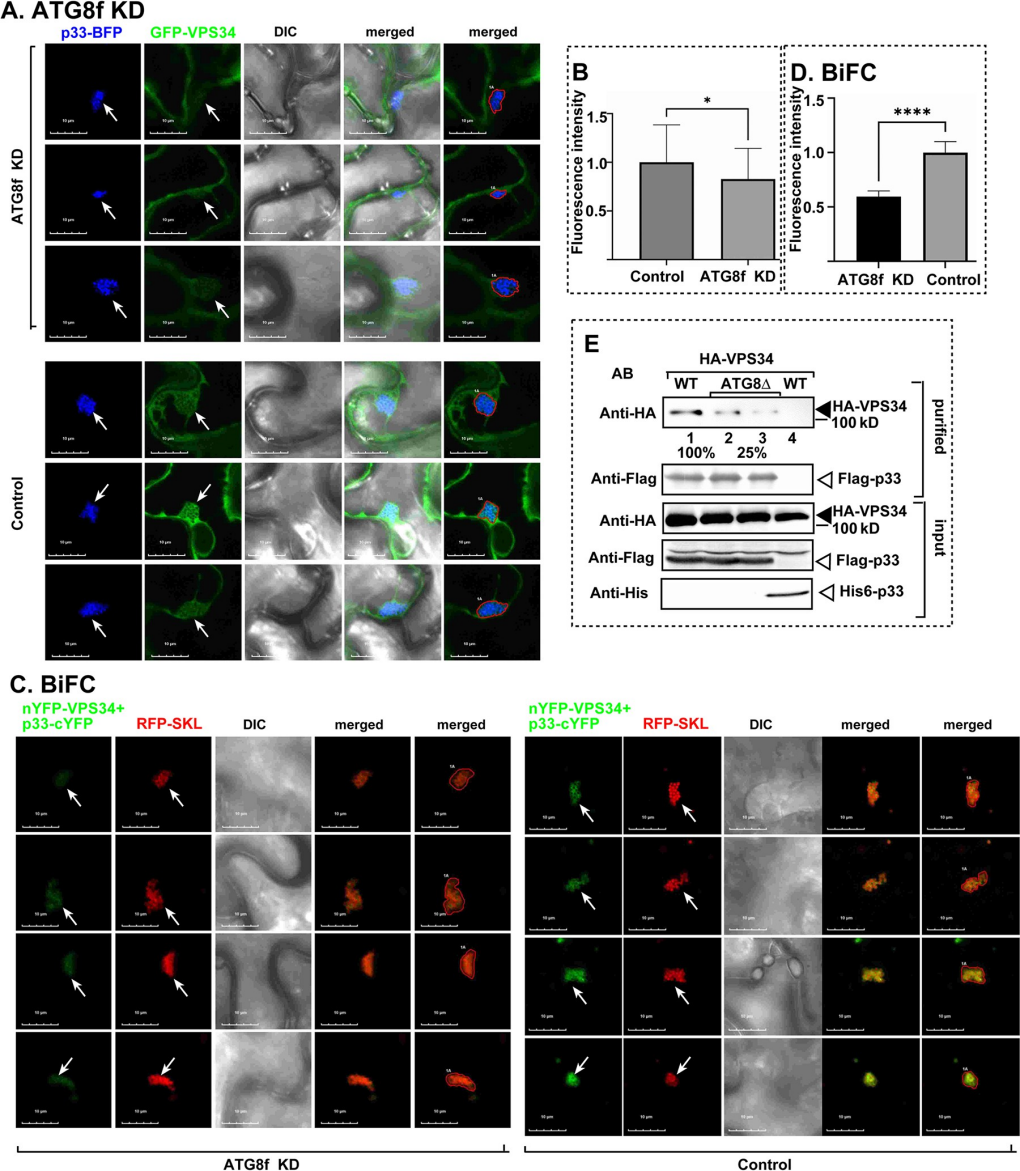
ATG8f promotes the enrichment of VPS34 PI3 kinase within VROs in *N*. *benthamiana* plants and yeast. (A) Confocal microscopy images show co-localization of TBSV p33-BFP replication protein and the AtVPS34-GFP, the PI(3) kinase, in ATG8f-silenced (ATG8f-KD) (top image) or TRV2-MBP control (bottom image) *N*. *benthamiana* cells. (B) Quantitative GFP fluorescence intensity values were measured for ~50 samples to calculate relative AtVPS34-GFP levels in VROs. The statistical analysis was performed using a t-test, and the results showed a significant difference between the two groups (p < 0.05). Each experiment was repeated. (C) BiFC-based assay was used to measure the effect of ATG8f knockdown on enrichment of VPS34 in VROs. *N*. *benthamiana* plants expressed TBSV p33-cYFP and AtVps34-nYFP in ATG8f-silenced (ATG8f-KD) (left panels) or TRV2-MBP control (right panels). (D) YFP fluorescence intensity values were quantified using Image J. YFP fluorescence intensity in control was arbitrarily set as 1. Error bars represent SD (n  =  60). Student t-test was used for statistical analysis of data (****P < 0.0001). (E) Co-purification of HA-VPS34 with Flag-p33 from either atg8Δ or WT (BY4741) yeasts. The membrane fraction of yeasts was detergent-solubilized and Flag-p33 was purified on the anti-Flag column, followed by elution. Western blotting was used to detect the co-purified HA-VPS34 using anti-HA antibody. Bottom panels: western blot of input HA-VPS34 in the total yeast extracts.

VPS34 PI(3) kinase is recruited to autophagic membranes to produce PI(3)P from PI phospholipid [83]. TBSV has been shown to hijack VPS34 into VROs to produce PI(3)P [44,53]. Usurping ATG8 and the selective autophagy pathway might be one of the ways for TBSV to efficiently co-opt the cytosolic VPS34. This idea was tested by co-expressing GFP-VPS34 and p33-BFP in ATG8f knockdown *N*. *benthamiana* infected with TBSV. Quantification of fluorescent signals in VROs by confocal microscopic analysis revealed reduction in VPS34 amount in ATG8f knockdown *N*. *benthamiana* in comparison with the control plants (Fig 10A and 10B). We also conducted BiFC experiments between nYFP-VPS34 and p33-cYFP in ATG8f knockdown plants. Interestingly we observed ~40% decrease in the BiFC signals in ATG8f knockdown versus control plants (Fig 10C and 10D). These findings support the role of ATG8f and the autophagy pathway in subversion of VPS34 PI3K by TBSV in *N*. *benthamiana*. Yeast has only one ATG8 gene and we used the null mutant (atg8Δ) to purify Flag-p33 from the detergent-solubilized membrane fraction. Interestingly, the co-purified HA-tagged VPS34 was ~4-fold less from atg8Δ yeast than from the WT yeast (Fig 10E, lanes 2–3 versus 1). This finding further supports the role of autophagy pathway in subversion of VPS34 PI3K by TBSV.

### Tombusvirus replication protein inhibits autophagic flux in *N*. *benthamiana*

**Fig 11:**
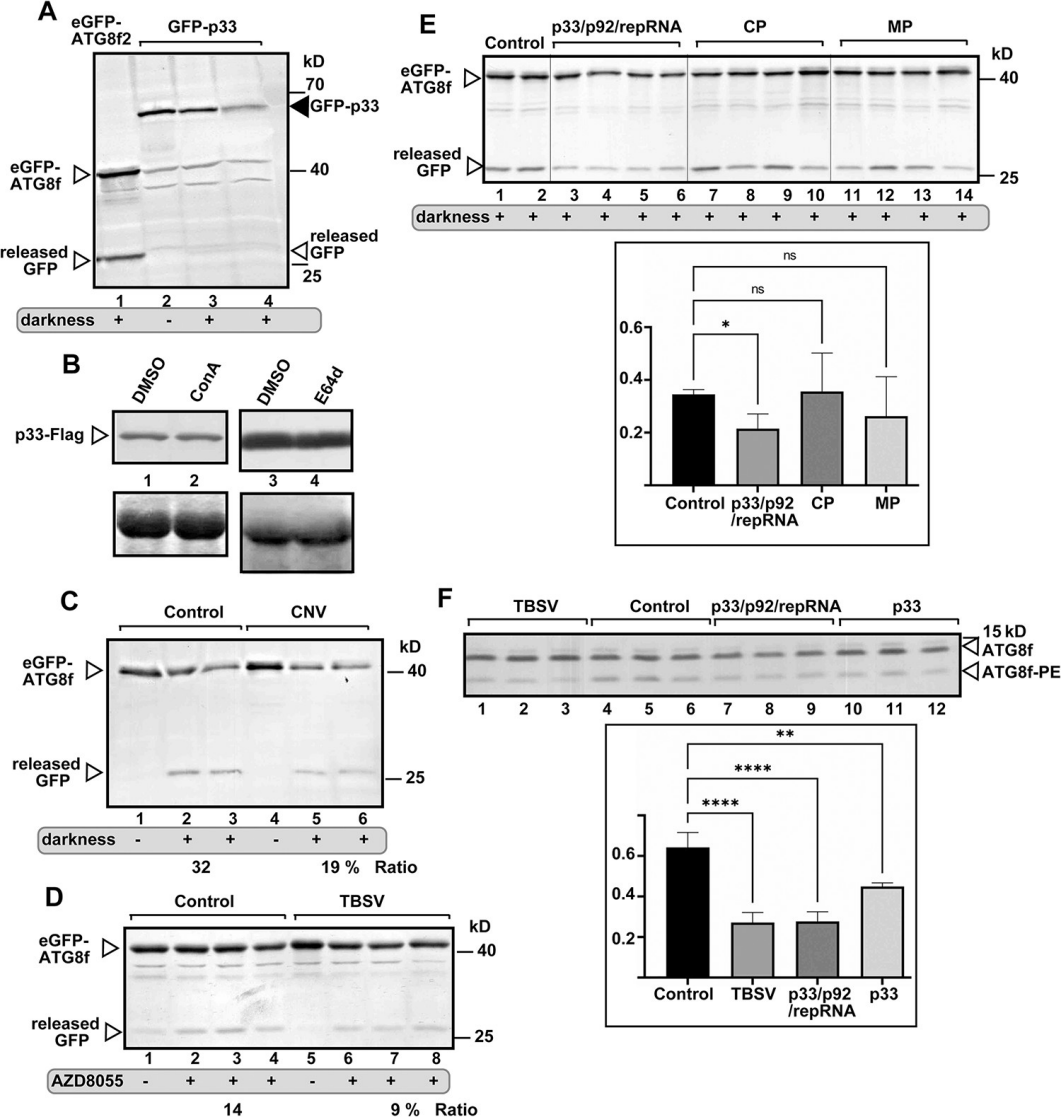
TBSV replication protein inhibits autophagic flux in *N*. *benthamiana*. (A) Agroinfiltrated *N*. *benthamiana* plants expressing GFP-p33 were exposed to darkness for 16 h to induce bulk autophagy (lanes 3 and 4) or kept under normal condition (lane 2). Total protein extracts were probed using western blot with anti-GFP antibody. *N*. *benthamiana* plants expressing GFP-ATG8f were used as a control (lane 1). The released ‘free’ GFP is marked with an arrowhead. (B) TBSV p33-Flag was expressed in *N*. *benthamiana* plants via agroinfiltration. 2.5 days latter, the agroinfiltrated leaves were treated with either 1 μM ConA or 100 μM E64d to inhibit autophagic degradation and samples were taken after 16 h. Western blotting was done with anti-Flag antibody. (C) Expression of GFP-ATG8f in *N*. *benthamiana* infected with CNV^20kstop^ (lanes 4–6) or pGD vector as the control (lanes 1–3) was done through agroinfiltration. At 1.5 dpi, two plants from each group were exposed to darkness for 16 h. Total protein extracts were probed using western blot with anti-GFP antibody. The released ‘free’ GFP is marked with an arrowhead. Autophagic flux was measured based on the ratio of GFP/GFP-ATG8f using Image J software. (D) Expression of GFP-ATG8f in *N*. *benthamiana* infected with TBSV (lanes 5–8) or pGD vector as the control (lanes 1–4) was done through agroinfiltration. The same leaves were infiltrated with 10 μM AZD8055 to induce bulk autophagy at 1.5 dpi or with 10 μM DMSO. Total protein extracts were obtained 8 h later and probed using western blot with anti-GFP antibody. The released ‘free’ GFP is marked with an arrowhead. Autophagic flux was measured based on the ratio of GFP/GFP-ATG8f using Image J software. (E) Expression of GFP-ATG8f (i) with the pGD vector as the control (lanes 1–2), (ii) with TBSV p33/p92/repRNA combination (lanes 3–6), (iii) with the TBSV coat protein (CP) (lanes 7–10) or (iv) with the movement protein (MP) (lanes 11–14) in *N*. *benthamiana* leaves was done through agroinfiltration. At 1.5 dpi all plants underwent 16 h darkness treatment. See more details in panel B above. The statistical analysis was performed using an ANOVA-test, and the results showed a significant difference between the control and ‘p33/p92/repRNA combination group’ (p < 0.05), while no significant difference between the control and CP or MP. (F) Expression of Flag-ATG8f (i) with the pGD vector as the control (lanes 4–6), (ii) with TBSV (lanes 1–3) (iii) with p33/p92/repRNA combination (lanes 7–9), or (iv) with TBSV p33 in *N*. *benthamiana* leaves was done through agroinfiltration. At 2 dpai, total protein extracts were obtained and probed using western blot with anti-Flag antibody. Autophagic flux was measured based on the ratio of ATG8f-PE/ATG8f using Image J software. The statistical analysis was performed using an ANOVA-test. ** (p < 0.01), **** (p < 0.0001). Each experiment was repeated.

Hijacking of ATG11 [87], NBR1, ATG8 and VPS34 and several other key autophagy components by tombusviruses into VROs might affect the autophagy pathway in infected cells. On the other hand, viral proteins are known to induce autophagy, leading to their degradation [103]. To test these possibilities, we expressed p33-GFP and followed its degradation in *N*. *benthamiana*. However, we did not detect the released ‘free’ GFP derived from p33-GFP in *N*. *benthamiana* (Fig 11A, lane 2). This suggests that p33 does not induce the complete autophagy pathway and p33 is not a substrate of autophagy under the conditions used. Accordingly, inhibition of autophagic degradation via ConA or E64d treatments did not alter p33 levels in *N*. *benthamiana* (Fig 11B). To test if induced bulk autophagy pathway could target p33 replication protein, we applied darkness treatment of plants [88] expressing p33-GFP. Interestingly, p33 was a poor autophagy substrate even under induced conditions in *N*. *benthamiana* (Fig 11A, lanes 3–4). In contrast, the control eGFP-ATG8f was a good autophagy substrate after darkness treatment of *N*. *benthamiana* expressing eGFP-ATG8f (Fig 11A, lane 1). To further test the effect of tombusviruses on the autophagy pathway, we infected *N*. *benthamiana* expressing eGFP-ATG8f with TBSV or the closely-related CNV. Interestingly, autophagic degradation of eGFP-ATG8f was not observed (Fig 11C, lanes 1 versus 4; Fig 11D, lanes 1 versus 5) suggesting that TBSV and CNV infections poorly induced the complete autophagy pathway in *N*. *benthamiana*. The basal level of autophagic flux was low in in *N*. *benthamiana* without darkness treatment (Fig 11C, lanes 1 versus 2 and 3; Fig 11D, lanes 1 versus 2–4). To test the possible effect of tombusviruses on the autophagy pathway, we induced autophagy via darkness or AZD8055-treatment [104] of *N*. *benthamiana* expressing eGFP-ATG8f and infected with either TBSV or CNV. Interestingly, reduced autophagic degradation of eGFP-ATG8f was observed in CNV or TBSV-infected versus non-infected plants (Fig 11C and 11D). To identify which TBSV proteins inhibit the autophagic degradation of eGFP-ATG8f, we expressed p33 and p92 replication proteins together with DI-72 replicon RNA, and separately, the coat protein and the movement protein of TBSV in *N*. *benthamiana* followed by darkness treatment. These experiments revealed that only the replication system inhibited the autophagic degradation of eGFP-ATG8f (Fig 11E).

We also performed another assay, which is based on lipidation of ATG8. Conjugation of ATG8 with PE is needed for the autophagy pathway [83]. The ATG8f lipidation assay revealed that expression of p33 replication protein or TBSV infection of *N*. *benthamiana* reduced ATG8f-PE conjugation by ~2-fold (Fig 11F), suggesting that TBSV infection interferes with the activation of autophagy to some extent.

**Fig 12:**
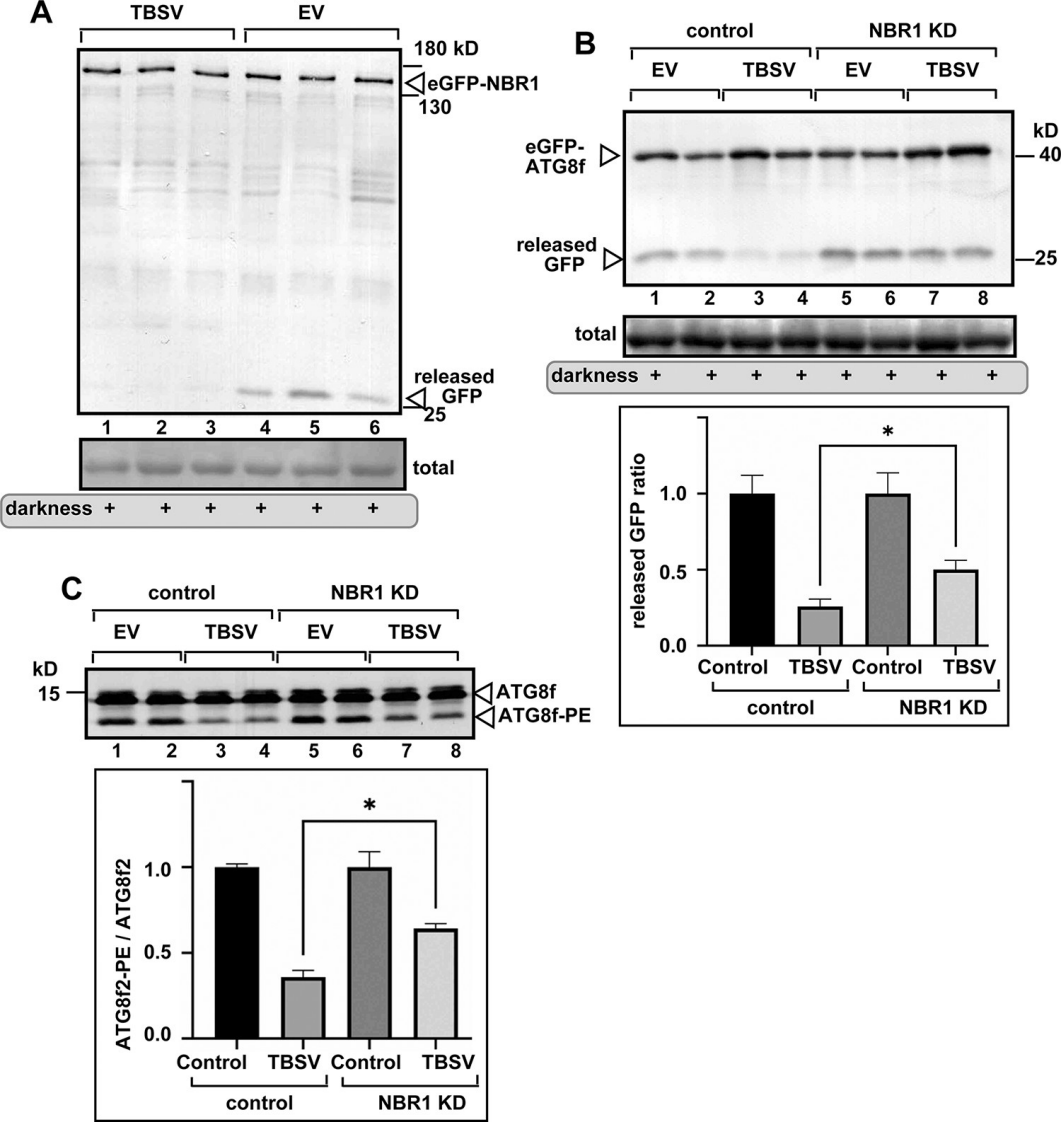
NBR1 affects inhibition of autophagic flux by TBSV in *N*. *benthamiana*. (A) Agroinfiltrated *N*. *benthamiana* plants co-expressing eGFP-NBR1 and either TBSV (lanes 1–3) or pGD vector control (lanes 4–6) were exposed to darkness for 16 h to induce bulk autophagy. Total protein extracts were obtained at 2 dpi and probed using western blot with anti-GFP antibody. The released ‘free’ GFP is marked with an arrowhead. (B) VIGS was used to obtain NBR1-silenced (NBR1-KD) or control (TRV2-MBP) *N*. *benthamiana* plants. Then, 10 days later, agroinfiltration was used to co-express GFP-ATG8f and either TBSV (lanes 3–4 and 7–8) or pGD vector control (lanes 1–2 and 5–6). Plants were exposed to darkness for 16 h to induce bulk autophagy. Total protein extracts were obtained and probed using western blot with anti-GFP antibody. The released ‘free’ GFP is marked with an arrowhead. Autophagic flux was measured based on the ratio of GFP/GFP-ATG8f using Image J software. We used the controls (1.0 value) for normalization of inhibition of autophagy by TBSV for each group. (C) VIGS was used to obtain NBR1-silenced (NBR1-KD) or control (TRV2-MBP) *N*. *benthamiana* plants. Then, 10 days later, agroinfiltration was used to co-express Flag-ATG8f and either TBSV (lanes 3–4 and 7–8) or pGD vector control (lanes 1–2 and 5–6). At 2 dpi, total protein extracts were obtained and probed using western blot with anti-Flag antibody. Autophagic flux was measured based on the ratio of ATG8f-PE/ATG8f using Image J software. We used the controls (1.0 value) for normalization of inhibition of autophagy by TBSV for each group. Each experiment was repeated. The statistical analysis was performed using an ANOVA-test. * (p < 0.05).

We also observed that autophagy-driven degradation of eGFP-NBR1 was inhibited in TBSV-infected *N*. *benthamiana* (Fig 12A). Autophagic flux was also inhibited by TBSV in NBR1 knockdown plants (Fig 12B). However, the inhibition of autophagic flux by TBSV was less pronounced than in the control plants, suggesting that inhibition of autophagic flux by TBSV requires NBR1. Similarly, ATG8f lipidation assay revealed that TBSV infection of NBR1 silenced *N*. *benthamiana* reduced ATG8f-PE conjugation to lesser extent than in control plants (Fig 12C). Altogether, these data suggest that hijacking of NBR1 by TBSV facilitates the inhibition of autophagy. It is likely that hijacking NBR1 (single gene) is more robust than the efficient hijacking of the ATG8 family (multiple genes) by the TBSV p33, which might explain why TBSV targets NBR1 selective autophagy receptor to inhibit selective autophagy.

### The co-opted ATG8f and NBR1 are present in biomolecular condensates associated with TBSV VROs

**Fig 13:**
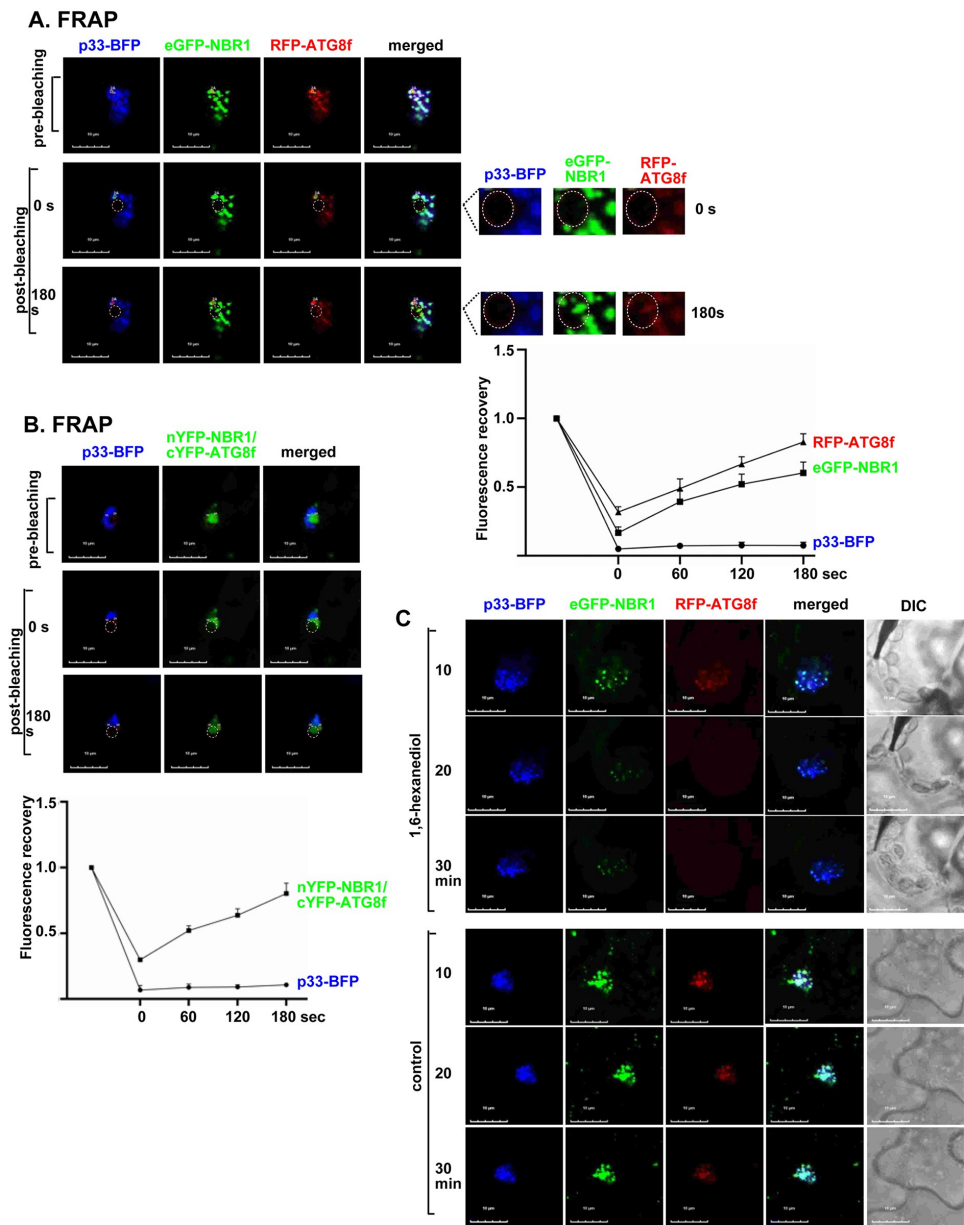
ATG8f and NBR1 are present in condensates associated with the TBSV VROs. (A) Agroinfiltrated *N*. *benthamiana* leaves co-expressing p33-BFP, eGFP-NBR1 and RFP-ATG8f were used in fluorescence recovery after photobleaching (FRAP) assay. Confocal images were taken before and after photobleaching for 180 sec. Time ‘0 s’ indicates the time of photobleaching. Scale bars represent 10 μm. Right panel: Quantification of FRAP signals of p33-BFP, eGFP-NBR1 and RFP-ATG8f in the photobleached area was done at the indicated time points after photobleaching. (B) FRAP analysis shows the fluorescence recovery of the BiFC signals after photobleaching within a single VRO induced by TBSV p33-BFP in a *N*. *benthamiana* cell. Agroinfiltrated *N*. *benthamiana* plants co-expressed nYFP-NBR1 and cYFP-ATG8f and p33-BFP. The graph shows the extent of fluorescence recovery in four individual VROs. (C) Agroinfiltrated *N*. *benthamiana* leaves co-expressing p33-BFP, eGFP-NBR1 and RFP-ATG8f for 1.5 d were treated with 10% 1,6-hexanediol or digitonin (control) for 30 min with 10 min intervals. Confocal images of four individual VROs were taken every 10 min. Scale bars represent 10 μm. Each experiment was repeated three times.

We frequently observed round shaped puncta formed by ATG8f and NBR1 associated with TBSV or CIRV VROs in confocal images of plant cells (Figs 1 and 5A). Frequent round shaped puncta were also found using BiFC based on ATG8f and NBR1 (Fig 5B) in TBSV-infected cells. It is known that LC3/ATG8 and p62/NBR1 form condensates under some conditions [105]. Therefore, we performed FRAP experiments on plant cells infected with TBSV. *N*. *benthamiana* also co-expressed eGFP-NBR1 and RFP-ATG8f and TBSV p33-BFP to mark the VROs. After photobleaching a portion of the VROs, we followed the recovery of fluorescent signals within the bleached area. As expected, the fluorescent signal for the membrane-bound p33-BFP did not recover (Fig 13A). This is likely due to limited movement of p33 anchored to the VRO membrane. On the other hand, the fluorescent signals for both eGFP-NBR1 and RFP-ATG8f in VROs were partially recovered after 180 sec (Fig 13A). The fluorescent signals for both eGFP-NBR1 and RFP-ATG8f, when they formed larger puncta associated with VROs, were also partially recovered after 180 sec (S9A Fig). The fluorescent signals were also partially recovered after 180 sec when RFP-ATG8f and eGFP-NBR1 were expressed separately in *N*. *benthamiana*, which also expressed p33-BFP (S9B–S9C Fig). These findings could be explained that a significant fraction of eGFP-NBR1 and RFP-ATG8f molecules are present in condensates within the VROs, which allow some internal molecular movement. Interestingly, fluorescent signals for other co-opted core autophagy proteins, such as ATG4, ATG5 and ATG101, were also partially recovered in 60–180 sec after photobleaching of portions of VROs (S10 Fig). The only exception was ATG1a, whose fluorescent signal was not recovered in VROs (S10 Fig). Thus, co-opted core autophagy proteins seem to be present and sequestered in condensate-like substructures in VROs.

We also performed FRAP experiments in combination with BiFC between nYFP-NBR1 and cYFP-ATG8f in plant cells infected with TBSV. The VROs were marked with p33-BFP. Interestingly, the BiFC signals within the VROs were partially recovered after 180 sec (Fig 13B). This suggests that NBR1 and ATG8f are present in the same condensates within the VROs. Treatment of condensates with 1,6-hexanediol disrupts weak hydrophobic interactions, which could dissolve condensates [106,107]. We found that treatment of plant cells infected with TBSV by 1,6-hexanediol partially dissolved the punctate structures containing NBR1 and ATG8f within the VROs (Fig 13C). On the contrary, 1,6-hexanediol treatment did not significantly affect the distribution of membrane-bound p33 within the VROs (Fig 13C). In the negative control experiments, treatment of plants cells with digitonin did not affect the punctate structures containing NBR1 and ATG8f within the VROs (Fig 13C). Overall, these results may suggest that a significant portion of NBR1 and ATG8f is sequestered within condensate-like substructures within the TBSV VROs.

## Discussion

### A complex interplay between selective autophagy and the tombusvirus replication protein supports VRO biogenesis

VRO biogenesis, which is the central step in tombusvirus replication, is a complex process depending on multiple interactions between tombusvirus and its host [25,47,108]. The master regulator and major driver of VRO biogenesis is the TBSV p33 replication protein, which subverts many co-opted host proteins and subcellular membranes [39,43,44,48,52]. The list of subverted proviral host factors now includes several autophagy proteins. VIGS-based knockdown of ATG8f and ATG5 core autophagy proteins and NBR1 selective autophagy receptor demonstrated the dependency of TBSV and the closely related CIRV replication on autophagy. We showed that the efficient recruitment of ATG8f and NBR1 by p33 replication protein into VROs contributed/enriched important lipids, such as PE and PI(3)P and VPS34 PI3K, to the VRO membranes. We have shown previously that these lipids are critical for spherules formation, the sites of viral replication, in host cells [39,44,45,53,109]. The autophagy membranes are enriched in PE and PI(3)P [100]. Interestingly, TBSV also hijacks Rab5-positive endosomes and retromer tubular vesicles to further increase PE and PI(3)P lipids in VRO membranes [39,44,45,53,109]. Subversion of multiple pathways and vesicles by TBSV to build VRO membranes seems to be necessary for robust and efficient viral replication. In addition, hijacking multiple pathways by TBSV could be advantageous in different hosts and cells, which could differ in various lipid resources.

Although TBSV usurps lipids from the autophagy membranes, we think it is unlikely that TBSV directly utilizes the double-membrane autophagy compartment for virus replication. Numerous previous publications showed that TBSV and other tombusviruses use either the limiting membrane of peroxisomes or the outer mitochondrial membrane for virus replication [14,47,48,110–115]. We propose that TBSV hijacks the autophagosome lipids/membranes during the early membrane expansion phase and repurposes the lipids for membrane proliferation in VROs.

### Subversion of ATG8f and NBR1 by p33 replication protein leads to reduced autophagy flux during virus replication

**Fig 14:**
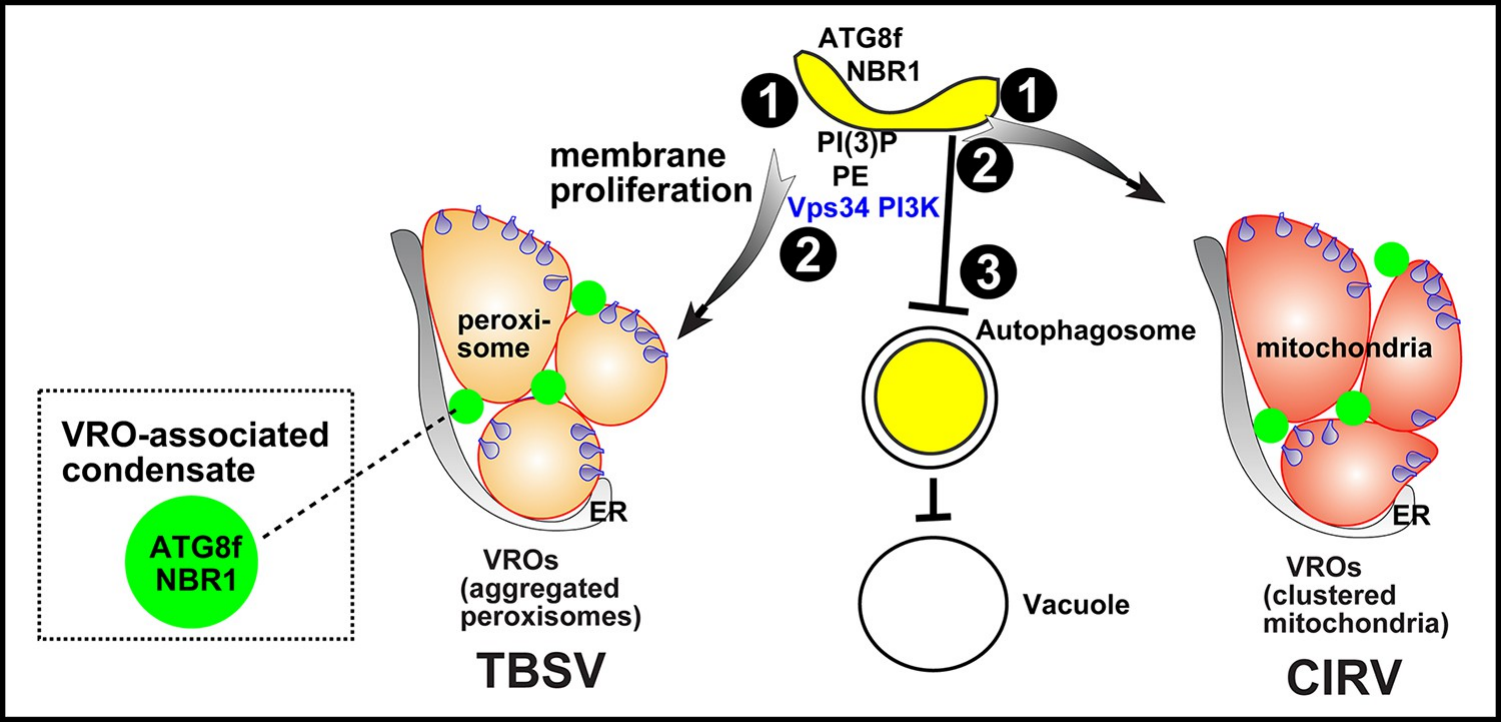
A model on subversion of selective autophagy for tombusvirus replication and inhibition of antiviral autophagy via sequestration of NBR1 and ATG8f in VRO-associated condensates. (#1) The TBSV p33 replication protein recruits ATG8f and NBR1 and other core autophagy proteins into VROs formed from clustered peroxisomes. The CIRV p36 replication protein performs comparable recruitment into VROs formed from clustered mitochondria. The co-opted ATG8f and NBR1 are sequestered and ‘trapped’ in condensates associated with VROs. (#2) Hijacking the autophagy pathway results in recruitment of VPS34 PI3 kinase and enrichment of phospholipids, such as PE and PI(3)P phosphoinositide needed for VROs biogenesis. (#3) Sequestration of ATG8f and NBR1 in VRO-associated condensate leads to inhibition of antiviral cellular autophagy, thus facilitating tombusvirus replication.

The emerging picture from this work is that, by usurping ATG8f and NBR1 and other autophagy components, such as ATG1a, ATG4, ATG5, ATG101 and SH3P2, TBSV inhibits the autophagic flux in plants. Accordingly, we found that p33 replication protein was not prone to degradation by autophagy. Moreover, p33 expression or TBSV infection inhibited the autophagic degradation of ATG8f and NBR1 under induced conditions (darkness or AZD8055 treatments of plants). In addition, TBSV moderately inhibited the lipidation of ATG8f, which is needed for the autophagy pathway [83]. Yet, we found that TBSV replication depended on the lipidation of ATG8f, based on (i) expression of RavZ protease that eliminated the lipidated form of ATG8 and inhibited TBSV replication; (ii) knockdown of ATG5, which is part of the ATG12-ATG5-ATG16 complex that lipidates ATG8s, also resulted in inhibition of TBSV replication. Thus, it seems that TBSV regulates the activity of the autophagy pathway to provide lipid/membrane resources for VRO biogenesis without turning on the antiviral activity of autophagy (Fig 14).

### ATG8f and NBR1 are sequestered by p33 replication protein into condensates associated with VROs during virus replication

How can TBSV inhibit the activation of the antiviral selective autophagy? We observed that a large fraction of ATG8f and NBR1 autophagy receptor was sequestered into small punctate structures within the VROs. FRAP analysis showed the partial fluorescent signal recovery for ATG8f and NBR1, but not for the membrane-anchored p33 replication protein, within the VROs. The fluorescent signal recovery for ATG8f also suggests that ATG8f is not lipidated within the puncta since membrane association would significantly limit the movement of ATG8f-PE within the VROs. We also found that the co-opted ATG8f and NBR1 interacted with each other within the puncta in VROs. Therefore, the emerging picture is that TBSV sequesters and “traps” the inactive ATG8f and NBR1 and other co-opted core autophagy proteins within condensates associated with VROs to regulate the autophagy pathway in *N*. *benthamiana* (Fig 14). Moreover, it seems that overexpression of NBR1 promoted the ‘trapping’ of ATG8f in large puncta within VROs.

We previously demonstrated that the TBSV p33 and the CIRV p36 replication proteins organize condensate formation by co-opted cytosolic proteins, such as glycolytic and fermentation enzymes and the proteasomal RPN11 protein interaction ‘hub’ within the VROs (ID#: BIORXIV/2023/550743). The p33 replication protein organized condensate substructure co-exists with the membranous substructure within the VROs. These substructures are likely hold together by the co-opted ER membranes and actin filaments, which form meshwork in the VROs. We propose that the co-opted autophagy proteins are also sequestered into the condensate substructure of the VROs, not inside the spherules (Fig 14). Altogether, sequestration of autophagy proteins in condensates associated with VROs might explain the inhibitory effect of TBSV infection on the autophagy pathway.

Interestingly, SARS-CoV-2 also induces condensates containing p62 (similar functions to the plant NBR1) and trapping selective ER-phagy receptors via the viral ORF8 protein [116]. The ORF8/p62 condensate formation leads to inhibition of ER-phagy and increased viral replication. In uninfected cells, the p62 condensate (also called p62 body) contains ubiquitinated cargoes and is degraded by autophagy to maintain cellular homeostasis [105,117–119]. Several negative-strand RNA viruses replicate in membraneless condensates formed by replication proteins and viral RNA in addition to co-opted host proteins [120–122]. Condensates formation is also observed during immune responses against infecting viruses [122–125]. Plant RNA virus movement depends on condensate formation [126]. Plant potyviruses induce RNA granules to facilitate virus replication [127]. Therefore, it seems that several RNA viruses exploit condensates for various viral functions.

Altogether, tombusviruses hijack the selective autophagy pathway in order (i) to enrich PE and PI(3)P lipids and VPS34 PI3K in VROs; (ii) to inhibit the antiviral autophagic flux; and (iii) to sequester and trap ATG8 and NBR1 in condensates associated with VROs. Overall, tombusviruses exploit autophagy for pro-viral functions. Other viruses also exploit autophagy for viral replication. Turnip mosaic virus co-opts NBR1, ATG8f and TIP1, which allows the viral replicase to associate with the tonoplast membrane to promote viral replication [65]. Zika virus and Dengue virus were shown to induce lipophagy and suppress ER-phagy by cleaving the ER-phagy receptor (FAM134B) [128,129]. Coronaviruses, enteroviruses and hepatitis C virus induce autophagy to hijack the double-membrane autophagosomes for replication or virion assembly [130]. The most frequent cases of viral exploitation of autophagy are based on viral protein-driven degradation of antiviral proteins, such as AGO1, suppressor of gene silencing 3 (SGS3) or SGS3/RDR6 bodies [79,131–133]. The emerging picture is that the interplay between autophagy and viruses is amazingly diverse, indicating forever lasting arms race between viruses and their hosts.

## Materials and methods

### Plant materials and plasmids

Wild type *N*. *benthamiana* plants were potted in soil and placed in growth room at 25°C under a 16 h light/8 h dark cycle. The nucleotide sequences of *N*. *benthamiana* genes NbATG5 (KX369397.1), NbATG8a (KX120976), NbATG8f2 (MG733107) and NbNBR1 (MG710800) were downloaded from NCBI GenBank. Total RNA extraction from *N*. *benthamiana* leaves was used for gene amplification. Reverse transcription was performed with Moloney Murine Leukemia Virus Reverse Transcriptase (M-MLV RT) (Promega) with Oligo(dT). Plasmids constructed (S1 Table) plasmids from previous works (S2 Table) and primers used in this study are listed in (S3 Table).

### Virus replication in plants

Virus induced gene silencing (VIGS) in *N*. *benthamiana* was performed as described in [134]. The cDNA of NbATG8f2 5’-terminal fragment of 205 bp in length (#8554: CGGGATCCATGGCTAAGAGCTCATTCAAG and #8576: CCGCTCGAGCTCAATTTGATTCTCTTGCG) was selected to insert into TRV2 vector, to generate pTRV2-NbATG8f, which was used for VIGS in *N*. *benthamiana*. Agrobacterium competent cells C58C1 were transformed with pTRV2-NbATG8f. *N*. *benthamiana* plants of 4-leaves stage were agroinfiltrated with pTRV1 and pTRV2-NbATG8f or pTRV2-cGFP as a control (OD_600_ 0.5). On the 9th day post agroinfiltration (dpai), upper leaves were agroinfiltrated to express CNV^20kstop^ or inoculated with TBSV or CIRV saps [135]. To determine RNA accumulation of TBSV, CNV, and CIRV, the inoculated leaves were collected at 2, 2.5 and 3 dpi, respectively. Total RNA extraction and northern blot analyses were performed as described previously [135]. The transcriptional accumulation of NbATG8 mRNA and internal reference control tubulin mRNA was determined by RT-PCR with primers oligo-d(T) (for RT), #8554 and #8555 (for PCR to detect NbATG8f) and #2859 and #2860 (for PCR to detect tubulin mRNA) [87,135].

To analyze the function of NbATG8a in tombusvirus replication, the 5’-terminal fragment of 224 bp in length (#7877: ACGCGGATCCATGGCCAAAAGCTCCTTCAAATTGG and #8575: CCGCTCGAGTTCTCAGCACTAAGCTTT) was selected for insertion into TRV2 vector, to generate pTRV2-NbATG8a, which was used for VIGS in *N*. *benthamiana*. Agrobacterium competent cells C58C1 were transformed with pTRV2-NbATG8a. *N*. *benthamiana* plants of 4-leaves stage were agroinfiltrated with pTRV1 and pTRV2-NbATG8a or pTRV2-cGFP as a control (OD_600_ 0.5). On the 9th day post agroinfiltration, the upper leaves were infiltrated with agrobacterium harboring virus infectious clone CNV20k^stop^ or inoculated with TBSV or CIRV sap. Total RNA extraction and northern blot analyses were described above.

To analyze the function of NbNBR1 in tombusvirus replication, the fragment of 400 bp in length (#8580: CGGGATCCATAGTGGGGAGGAGAAGG and #8581: CCGCTCGAGTGACCCCTTTTATATGGG) was inserted into the TRV2 vector to generate pTRV2-NbNBR1, which was used for VIGS in *N*. *benthamiana*. Agrobacterium competent cells C58C1 were transformed with pTRV2-NbNBR1. *N*. *benthamiana* plants of 4-leaves stage were agroinfiltrated with pTRV1 and pTRV2-NbNBR1 or pTRV2-cGFP as a control (OD_600_ 0.5). On the 9th day post agroinfiltration, the upper leaves were inoculated with TBSV or CIRV sap. Total RNA extraction and northern blot analyses were described above.

To analyze the function of NbATG5 in tombusvirus replication the NbATG5 3’-terminal fragment of 384 bp in length (#7749: CCGCTCGAGCTTACATAAACAGACCTG and #7750: CGGGATCCATATGGTGATGGGTTCTTG) was selected for insertion into TRV2 vector, to generate pTRV2-ATG5, which was used for VIGS in *N*. *benthamiana*. *N*. *benthamiana* plants of 4-leaves stage were agroinfiltrated with pTRV1 and pTRV2-NbATG5 or pTRV2-cGFP as a control (OD_600_ 0.5). On the 9th day post agroinfiltration, the upper leaves were infiltrated with agrobacterium harboring virus infectious clone CNV20k^stop^ or inoculated with TBSV or CIRV sap. Total RNA extraction and northern blot analyses were described above.

### Confocal laser microscope studies in plants

To analyze the subcellular localization of NbATG8f, NbATG8a and NbNBR1 in the presence or absence of viral components in *N*. *benthamiana* leaves, pGD-35S-p33-BFP, pGD-35S-C36-BFP, pGD-35S-GFP-NbATG8f, pGD-35S-GFP-NbATG8a, pGD-35S-GFP-NbNBR1, pGD-35S-RFP-SKL (as a peroxisome marker) and pGD-35S-RFP-AtTim21 (as a mitochondrial marker) [39] were transformed into agrobacterium strain C58C1. Then agrobacterium cultures with different combinations were infiltrated into *N*. *benthamiana* leaves, followed by virus inoculation with TBSV or CIRV sap at 16 h post agroinfiltration. At 2.5 dpai, the agroinfiltrated leaves were subjected to confocal laser microscopy with Olympus FV3000.

To detect interactions between NbATG8f and NbNBR1 and TBSV p33 or CIRV p36 replication proteins, bimolecular fluorescence complementation (BiFC) assay was performed. pGD-35S-T33-cYFP, pGD-35S-C36-cYFP, pGD-35S-C-cYFP (as a negative control), pGD-35S-nYFP-NbATG8f, pGD-35S-nYFP-NbNBR1, pGD-35S-nYFP-MBP (as a negative control), pGD-35S-RFP-SKL (as a peroxisome marker) and pGD-35S-RFP-AtTim21 (as a mitochondrial marker) were transformed into agrobacterium strain C58C1. The Agrobacterium transformants with different combinations were used to infiltrate *N*. *benthamiana* leaves. At 2.5 dpai, the agroinfiltrated leaves were subjected to confocal laser microscopy.

To test if NbATG8f interacts with NbNBR1 in the presence or absence of TBSV p33, *N*. *benthamiana* leaves were co-agroinfiltrated with pGD-35S-nYFP-NbNBR1, pGD-35S-NbATG8f-cYFP, pGD-35S-BFP-p33 and pGD-35S-RFP-SKL. The agroinfiltrated *N*. *benthamiana* leaves were subjected to confocal laser microscopy at 2.5 dpai.

To observe the subcellular distribution of PE (phosphatidylethanolamine) in plant mesophyll protoplasts, first we silenced ATG8f and NBR1, respectively, in *N*. *benthamiana* via VIGS as above for 10 d. Then, the top leaves were agroinfiltrated to express p33-RFP (pGD-p33-RFP), p19 (pGD-p19) with or without RavZ expression [52]. Protoplasts were isolated from the agroinfiltrated leaves of *N*. *benthamiana* 2 d later. The protoplasts were fixed with 3.7% paraformaldehyde and stained with duramycin as described previously [45].

To observe the subcellular distribution of PI(3)P (Phosphatidylinositol 3-phosphate) in plant mesophyll protoplasts, protoplasts were isolated from *N*. *benthamiana* leaves 2 dpi after the agroinfiltration with pGD-p33-BFP, pGD-p19 with or without RavZ. The protoplasts were fixed with 3.7% paraformaldehyde and incubated with purified anti-PI(3)P mouse antibody (Echelon Biosciences Inc. Cat#Z-P003) as described previously [101]. RFP-2xFYVE was used as a PI(3)P biosensor to visualize PI(3)P distribution upon virus replication in plant leaves [44].

To detect interaction of AtVps34 with TBSV p33 replication protein based on BiFC assay, plasmids pGD-35S-T33-cYFP, pGD-35S-nYFP-AtVps34, pGD-35S-RFP-SKL (as a peroxisome marker) were transformed separately into agrobacterium strain C58C1. Mixed agrobacterium cultures were used to infiltrate ATG8f-silenced and control leaves separately (see above). At 2 dpai, the agroinfiltrated leaves were subjected to confocal laser microscopy using the same laser power.

### Protein proximity-labeling assay in plants

To detect the close proximity of p33 replication protein and NbATG8f in plants, *N*. *benthamiana* leaves were agroinfiltrated with pGD-p33-His-BirA (OD_600_ 0.4), pGD-His-Avi-NbATG8f (OD_600_ 0.4) and pGD-P19 (OD_600_ 0.1) [87,94]. Agroinfiltration with pGD-P33-Myc (OD_600_ 0.4) was used as a control. The infiltrated leaves at 3 dpai were further infiltrated with 50 μM Biotin. Then the infiltrated leaves, after 40 minutes of biotin treatment, were harvested and subjected to protein extraction. Biotinylated His-Avi-NbATG8f protein was detected by western-blotting using Strep-AP [87].

### Protein purification from yeast

*S*. *cerevisiae* strain BY4741 (MATa his3Δ1 leu2Δ0 met15Δ0 ura3Δ0) was purchased from Open Biosystems and stored in a -80°C refrigerator. Yeast strain atg8Δ was generated as described previously from the BY4741 parental strain by replacing ATG8 ORF with a hphNT1 cassette sequentially using homologous recombination [136].

For co-purification of VPS34 protein with the TBSV p33/p92 replication proteins from yeasts, plasmids HpGBK-CUP1-Hisp33/Gal-DI-72 and LpGAD-CUP1-His92 (as a control) or HpGBK-CUP1-Flagp33/Gal-DI-72 and LpGAD-CUP1-Flag92 were co-transformed with UpYES-HA-ScVPS34 into yeast strain BY4741 and atg8Δ. All transformed yeasts were pre-grown in SC-ULH^−^ media supplemented with 2% glucose and 100 μM BCS at 29°C for 16 h. Then, yeast cultures were resuspended in SC-ULH^−^ medium supplemented with 2% galactose and 100 μM BCS and grown at 23°C for 24 h, followed by culturing yeast cells in (N^−^) SC-ULH^−^ medium supplemented with 2% galactose and 50 μM CuSO_4_ at 23°C for 6 h. Finally, yeast pellets were harvested after washing twice with PBS buffer and proteins were Flag-affinity purified as described previously [137].

### Protein purification from *N*. *benthamiana*

Various combinations of expression vectors were co-agroinfiltrated into *N*. *benthamiana* leaves and samples were harvested at 2.5 days post agroinfiltration and ground in a cooled mortar in GEN buffer (10% [v/v] glycerol, 25 mM Tris-HCl, pH 7.5, 1 mM EDTA, 150 mM NaCl, 10 mM DTT, 0.5% [v/v] Triton X-100 and protease inhibitor cocktail). The supernatants were incubated with anti-Flag M2 affinity agarose (Sigma-Aldrich) in Bio-spin chromatography columns (Bio-rad) for 2 h at 4°C on a rotator, followed by washing with the washing buffer (10% [v/v] glycerol, 25 mM Tris-HCl, pH 7.5, 1 mM EDTA, 150 mM NaCl, 1mM DTT and 0.1% [v/v] Triton X-100). Elution of purified proteins was as described above [137].

### Pull-down assay

For expression of MBP or MBP-tagged p33C (C-terminal portion of the TBSV p33) and p33C mutant, plasmids pMALc-2X, pMALc-2X-T33C, pMALc-2X-T33C-F32A/V35A and pMALc-2X-T33C-37-82 were transformed into Epicurion Bl21-codon-plus (DE3)-R1L cells, followed by IPTG induction. After sonication, 500 μl lysates were incubated with 30 μl amylose resin (NEB) in Bio-spin chromatography columns for 2 h at 4°C, followed by five times washing with column buffer [138]. The amylose columns containing the MBP or MBP-tagged p33C or p33C mutants were then incubated with 3 μg of the purified GST-His_6_-NbATG8f for 2 h at 4°C. Then, the washed beads were incubated in 1× SDS loading buffer for 15 min at 85°C. The MBP-tagged proteins were analyzed by SDS-PAGE followed by coomassie staining and GST-His_6_-tagged proteins were separated by SDS-PAGE for protein gel blot analysis with anti-His antibody [138].

### Monitoring autophagic activity in plants

To measure the autophagic activity in plants during TBSV replication, we employed the free-GFP release assay [98]. *N*. *benthamiana* leaves were agroinfiltrated with pGD-eGFP-ATG8f (OD_600_ 0.4) and different agrobacterium combinations. The infiltrated leaves at 1.5 dpai were exposed to darkness for 16 h or infiltrated with 10 μM AZD8055 [104]. Then leaf samples were harvested and subjected to protein extraction. Free GFP was detected by western blotting using anti-GFP antibody.

NbATG8f-II (PE conjugation) detection assay was performed to detect the autophagic activity [98,139]. *N*. *benthamiana* leaves were agroinfiltrated with pGD-Flag-ATG8f (OD_600_ 0.3) and different agrobacterium combinations. At 2 dpi, leaf samples were harvested and subjected to protein extraction. Total protein was loaded onto 15% polyacrylamide gels containing 6 M urea to separate ATG8f-PE from unconjugated ATG8f. NbATG8f-I and NbATG8f-II forms were detected by western blotting using anti-Flag antibody.

The effect of autophagic degradation on p33 protein levels was tested by using autophagic inhibitors. After 2 days post-agroinfiltration to express p33-Flag in whole *N*. *benthamiana* leaves, half-leaf inhibitor treatment was conducted. In brief, either 1 μM ConA or 100 μM E64d was infiltrated at the left side of each *N*. *benthamiana* leaf and the right side of leaves was treated with 0.5% DMSO as a control. After treatment for 12 h, leaf samples were harvested for total protein extraction and western blot analysis with anti-Flag antibody was performed as above.

### FRAP assay

FRAP assays were performed using an Olympus FV3000 confocal microscope [140]. *N*. *benthamiana* leaves were agroinfiltrated with different agrobacterium combinations. After 48 h, target region was bleached for 10 s at intensity of 30% at 405 nm laser. Fluorescence recovery was recorded over 180 s with 60 s interval. Mean fluorescence intensity was quantified by Image J and the values were normalized to background.

We applied 1,6 Hexanediol to characterize the VRO-associated condensates [141,142]. *N*. *benthamiana* leaves were agroinfiltrated with different agrobacterium combinations. After 48 h, leaves were infiltrated with 10% 1,6 hexanediol (dissolved in 10 μg/ml digitonin) or digitonin (10 μg/ml) as negative control. Images of VROs were recorded over 30 min with 10 min intervals.

### Quantification and statistical analysis

Statistical analysis was performed using GraphPad Prism 8 software. Details of the statistical tests and sample sizes are provided in the figure legends. Results with a p value of less than 0.05 were considered statistically significant, while results with a p value greater than 0.05 were considered statistically non-significant (ns).

## Supporting information

S1 Fig Role of ATG8a and ATG8i in tombusvirus replication in *N*. *benthamiana*. (A) Confocal microscopy images show co-localization of CIRV p36-RFP replication protein and BFP-ATG8a within VROs in *N*. *benthamiana* leaves. See further details in Fig 1A. (B) Confocal microscopy images show co-localization of TBSV p33-BFP replication protein and GFP-ATG8i within VROs consisting of clustered peroxisomes, marked by RFP-SKL peroxisomal matrix marker in *N*. *benthamiana* leaves. The expression of these proteins, driven by the 35S promoter, was achieved through co-agroinfiltration into N. *benthamiana* leaves. The plant leaves were TBSV-infected as shown. Scale bars represent 10 μm. (C) Confocal microscopy images show co-localization of CIRV p36-BFP replication protein and the GFP-ATG8i within VROs consisting of clustered mitochondria, marked by RFP-AtTim21 mitochondrial marker in *N*. *benthamiana* leaves. See further details in panel A. (D) BiFC experiments revealed interaction of nYFP-ATG8i with both TBSV p33-cYFP and CIRV p36-cYFP replication proteins. The merged images show co-localization of RFP-SKL (top panel) or RFP-AtTim21 (bottom panel) with the BiFC signals, indicating that the interactions take place in VROs. Scale bars represent 10 μm. (E) Top panel: The accumulation of the TBSV genomic (g)RNA in ATG8i-silenced (ATG8i KD) *N*. *benthamiana* plants at 2 dpi is shown in an ethidium-bromide stained gel. Inoculation with TBSV sap was done 10 days after silencing of ATG8i expression. TRV vectors carrying either ATG8i or 3′-terminal GFP (as a control) sequences were used to induce VIGS. Second panel: RT-PCR analysis of ATG8i mRNA level in the silenced and control plants. Third panel: RT-PCR analysis of tubulin mRNA level in the silenced and control plants. The bottom two panels were from the same gels, respectively. Each experiment was repeated.
(TIF)

S2 Fig Co-localization of the minus-strand of replicon RNA with ATG8a in VROs in *N*. *benthamiana* leaves infected with CNV. The (-)replicon RNA [(-)repRNA-MS2hp)] is based on DI-72 replicon RNA. However, it also carries 6 copies of the 19 nt long hairpin sequence from the MS2 phage, which is specifically recognized by the RFP-tagged MS2-CP (coat protein). Note that the hairpin structures form only on the minus strand RNAs, which are made during replication. RFP-MS2-CP is localized to the nucleus in the absence of replication of (-)repRNA-MS2hp (no helper CNV infection). Confocal microscopy images show the co-localization of the minus strand (-)repRNA-MS2hp, which is the replication intermediate, with GFP-ATG8a within the VRO. The VRO is marked by TBSV p33-BFP. Expression of the above proteins and the (-)repRNA-MS2hp was from 35S promoter via co-agroinfiltration into *N*. *benthamiana* leaves also infected with CNV to provide the replication proteins. Scale bars represent 10 μm. The experiment was repeated.
(TIF)

S3 Fig Recruitment of core ATG proteins by the TBSV p33 replication protein into VROs in *N*. *benthamiana*. Confocal microscopy images show co-localization of TBSV p33-BFP replication protein and ATG proteins (GFP-AtATG4, GFP-NbATG5, GFP-NbATG1a, GFP-NbATG101, and GFP-AtSH3P2) within VROs consisting of clustered peroxisomes, marked by RFP-SKL peroxisomal matrix marker in *N*. *benthamiana* leaves. Control experiments included the localization of the above ATG proteins in the absence of TBSV p33-BFP replication protein. The expression of these proteins, driven by the 35S promoter, was achieved through co-agroinfiltration into N. *benthamiana* leaves. Scale bars represent 10 μm. Each experiment was repeated.
(TIF)

S4 Fig Additional experiments to confirm interactions between tombusvirus replication proteins and ATG8. (A) Co-purification of His_6_-NbATG8a with TBSV Flag-p33 and Flag-p92^pol^ replication proteins from subcellular membranes of yeast. Top two panels: western blot analysis of co-purified His_6_-NbATG8a detected with anti-His antibody, while Flag-p33 was detected with anti-Flag antibody. The negative control was from yeast expressing His_6_-p33 purified on a Flag-affinity column (lane 1). Samples were cross-linked with formaldehyde. Bottom two panels: western blot of input His_6_-NbATG8a and Flag-p33 in the total yeast extracts. (B) Pulldown assay including GST-His_6_-ATG8f and the MBP-tagged TBSV p33 replication protein. Note that we used the soluble N-terminal region (1–82 aa) of TBSV p33, which contains the predicted AIM1 motif (NI**F**QL**V**). The F and V amino acids were mutated to As to eliminate the canonical AIM1 in p33-1-82AA (S4B Fig, lane 2). Top panel: western blot analysis of the eluted MBP-p33 protein was performed with anti-MBP antibody. The negative control was the MBP (lane 4). Middle panel: Western blot analysis of the eluted GST-His_6_-ATG8f from the GST column. Bottom panels: Coomassie-blue stained SDS-PAGE of affinity-purified MBP-p33 proteins and MBP from *E*. *coli*. (C) The split ubiquitin-based MYTH assay was used to test binding between either GST-His_6_-ScATG8 or GST-His_6_-NbATG8a and TBSV p33 protein in yeast. The bait p33 was co-expressed with the shown prey proteins. The bait p33 and the empty prey vector (NubG) were used as negative controls, and the bait p33 and ScSSA1 as a positive control, respectively. The right panel shows the interactions, whereas the left panel demonstrates that comparable amounts of yeasts were used for these experiments.
(TIF)

S5 Fig Interactions between TBSV p33 replication protein and core ATG proteins in *N*. *benthamiana*. (A) Protein proximity-labeling was performed with biotin in *planta*. *N*. *benthamiana* leaves were agroinfiltrated to express p33 replication protein, which was fused to BirA biotin ligase, and Avi-tagged ATG proteins (Avi-ATG4, Avi-ATG5, Avi-ATG101 and Avi-NBR1). Biotin treatment lasted for 40 min. The image shows the western blot analysis of the biotinylated Avi-ATG proteins and Avi-NBR1 detected with streptavidin-conjugated AP in total protein extracts. The experiment was repeated. (B) Co-purification of ATG proteins (His_6_-ATG4, His_6_-ATG5, or His_6_-ATG101) with TBSV Flag-p33 replication protein from *N*. *benthamiana* plants. Top two panels: western blot analysis of co-purified His_6_-ATG proteins detected with anti-His antibody, whereas Flag-p33 was detected with anti-Flag antibody. Bottom panel: western blot of total His_6_-ATG proteins in the total protein extracts.
(TIF)

S6 Fig SH3P2 interacts with TBSV p33 replication protein and ATG8f within VROs in *N*. *benthamiana*. (A) Interaction between TBSV p33-cYFP replication protein and the nYFP-SH3P2 protein was detected by BiFC. The merged images show the co-localization of RFP-SKL with the BiFC signals, indicating that the interaction between p33 replication protein and SH3P2 occurs in VROs in clustered peroxisomal membranes. Scale bars represent 10 μm. (B) BiFC assay was conducted to demonstrate the interaction between nYFP-SH3P2 and cYFP-ATG8f proteins within the p33-BFP-positive VROs. The expression of proteins was achieved via co-agroinfiltration into *N*. *benthamiana* leaves. Scale bars represent 10 μm. Each experiment was repeated three times.
(TIF)

S7 Fig RT-PCR analysis of mRNA levels of ATG8 family members in ATG8f silenced plants. The semi-quantitative RT-PCR analysis was conducted on the same set of plant samples to assess the effectiveness of ATG8f silencing. The second panel shows comparable mRNA levels of ATG8a and bottom panel for ATG8i, indicating selective gene silencing of ATG8f. Top panel: The RT-PCR analysis of tubulin mRNA level in the ATG8f-silenced (lanes 4–6) and control (lanes 1–3) plants. The panels were from the same gels, respectively. The experiment was repeated.
(TIF)

S8 Fig Nonlipidated ATG8f is recruited by TBSV p33 replication protein into VROs. Confocal microscopy images show co-localization of RFP-ATG8f and p33-BFP in *N*. *benthamiana* cells. The leaves either expressed GFP-RavZ effector (top panel), or pGD vector as control (bottom panel). The VROs are marked with arrows. See further details in Fig 6B. The experiment was repeated.
(TIF)

S9 Fig FRAP analysis of ATG8f and NBR1 shows their presence in condensates associated with the TBSV VROs. (A) Agroinfiltrated *N*. *benthamiana* leaves co-expressing p33-BFP, eGFP-NBR1 and RFP-ATG8f were used in fluorescence recovery after photobleaching (FRAP) assay. Confocal images were taken before and after photobleaching for 180 sec. Time ‘0 s’ indicates the time of photobleaching. Note that we selected a large punctate structure for photobleaching. Scale bars represent 10 μm. (B-C) Agroinfiltrated *N*. *benthamiana* leaves co-expressing p33-BFP and RFP-ATG8f (B) or p33-BFP and RFP-NBR1 (C) were used in a FRAP assay. Confocal images were taken before and after photobleaching for 180–360 sec. Time ‘0 s’ indicates the time of photobleaching. Scale bars represent 10 μm. Quantification of FRAP signals of p33-BFP, RFP-ATG8f and RFP-NBR1 in the photobleached area was done at the indicated time points after photobleaching. Confocal images of four individual VROs were taken. Scale bars represent 10 μm. Each experiment was repeated three times.
(TIF)

S10 Fig FRAP analysis of core ATG8 proteins in the TBSV VROs. (A) Agroinfiltrated *N*. *benthamiana* leaves co-expressing p33-BFP, and one of following: RFP-ATG4, BFP-ATG1a, RFP-ATG101 and RFP-ATG5 were used in FRAP assays. Confocal images were taken before and after photobleaching for 60–180 sec. Time ‘0 s’ indicates the time of photobleaching. Quantification of FRAP signals of p33-BFP, RFP-ATG4, BFP-ATG1a, RFP-ATG101 and RFP-ATG5 in the photobleached area was done at the indicated time points after photobleaching. Confocal images of four individual VROs were taken. Each experiment was repeated three times.
(TIF)

S1 Table Plasmids constructed in this study. (DOCX)

S2 Table Plasmids described in previous study. (DOCX)

S3 Table Primers used in this study. (DOCX)
