# Supplementary figures and images for: Subversion of selective autophagy for the biogenesis of tombusvirus replication organelles inhibits autophagy
Source: PLoS Pathog. 2024 Mar 14;20(3):e1012085. doi: 10.1371/journal.ppat.1012085 (PMC10965100; doi:10.1371/journal.ppat.1012085)

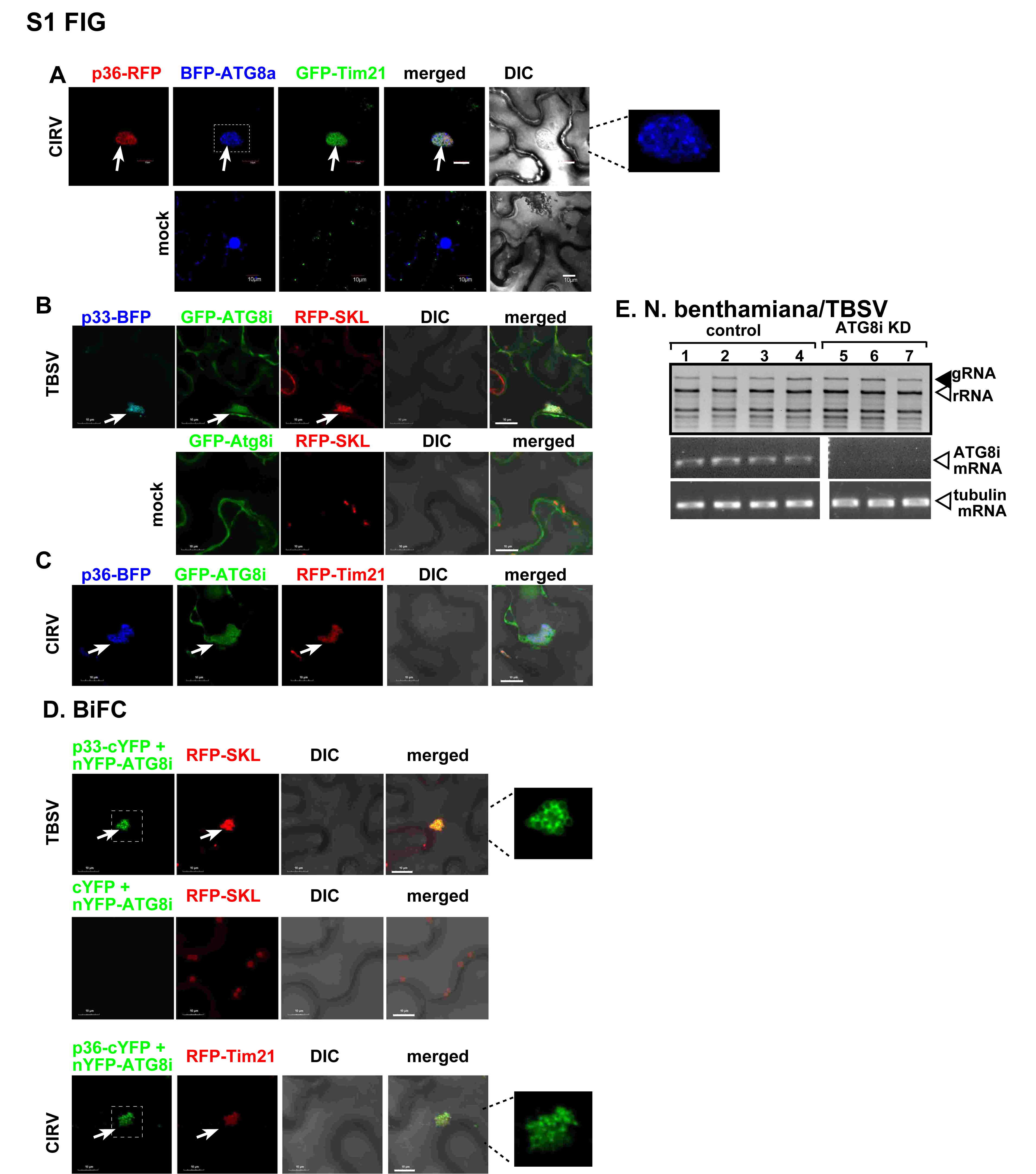

Supplement: S1 Fig — (A) Confocal microscopy images show co-localization of CIRV p36-RFP replication protein and BFP-ATG8a within VROs in N. benthamiana leaves. See further details in Fig 1A. (B) Confocal microscopy images show co-localization of TBSV p33-BFP replication protein and GFP-ATG8i within VROs consisting of clustered peroxisomes, marked by RFP-SKL peroxisomal matrix marker in N. benthamiana leaves. The expression of these proteins, driven by the 35S promoter, was achieved through co-agroinfiltration into N. benthamiana leaves. The plant leaves were TBSV-infected as shown. Scale bars represent 10 μm. (C) Confocal microscopy images show co-localization of CIRV p36-BFP replication protein and the GFP-ATG8i within VROs consisting of clustered mitochondria, marked by RFP-AtTim21 mitochondrial marker in N. benthamiana leaves. See further details in panel A. (D) BiFC experiments revealed interaction of nYFP-ATG8i with both TBSV p33-cYFP and CIRV p36-cYFP replication proteins. The merged images show co-localization of RFP-SKL (top panel) or RFP-AtTim21 (bottom panel) with the BiFC signals, indicating that the interactions take place in VROs. Scale bars represent 10 μm. (E) Top panel: The accumulation of the TBSV genomic (g)RNA in ATG8i-silenced (ATG8i KD) N. benthamiana plants at 2 dpi is shown in an ethidium-bromide stained gel. Inoculation with TBSV sap was done 10 days after silencing of ATG8i expression. TRV vectors carrying either ATG8i or 3′-terminal GFP (as a control) sequences were used to induce VIGS. Second panel: RT-PCR analysis of ATG8i mRNA level in the silenced and control plants. Third panel: RT-PCR analysis of tubulin mRNA level in the silenced and control plants. The bottom two panels were from the same gels, respectively. Each experiment was repeated. (TIF) [file ppat.1012085.s001.tif]

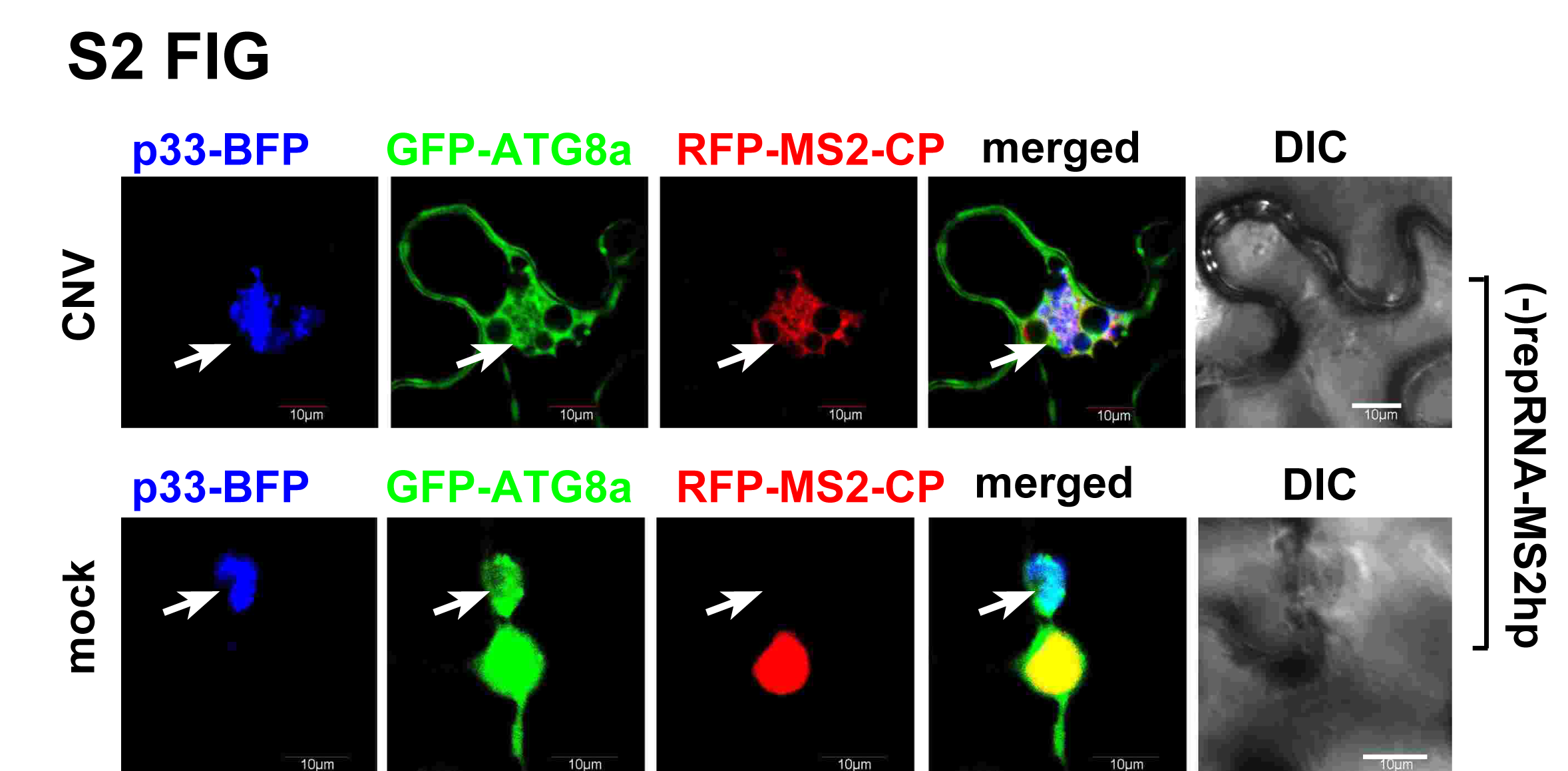

Supplement: S2 Fig — The (-)replicon RNA [(-)repRNA-MS2hp)] is based on DI-72 replicon RNA. However, it also carries 6 copies of the 19 nt long hairpin sequence from the MS2 phage, which is specifically recognized by the RFP-tagged MS2-CP (coat protein). Note that the hairpin structures form only on the minus strand RNAs, which are made during replication. RFP-MS2-CP is localized to the nucleus in the absence of replication of (-)repRNA-MS2hp (no helper CNV infection). Confocal microscopy images show the co-localization of the minus strand (-)repRNA-MS2hp, which is the replication intermediate, with GFP-ATG8a within the VRO. The VRO is marked by TBSV p33-BFP. Expression of the above proteins and the (-)repRNA-MS2hp was from 35S promoter via co-agroinfiltration into N. benthamiana leaves also infected with CNV to provide the replication proteins. Scale bars represent 10 μm. The experiment was repeated. (TIF) [file ppat.1012085.s002.tif]

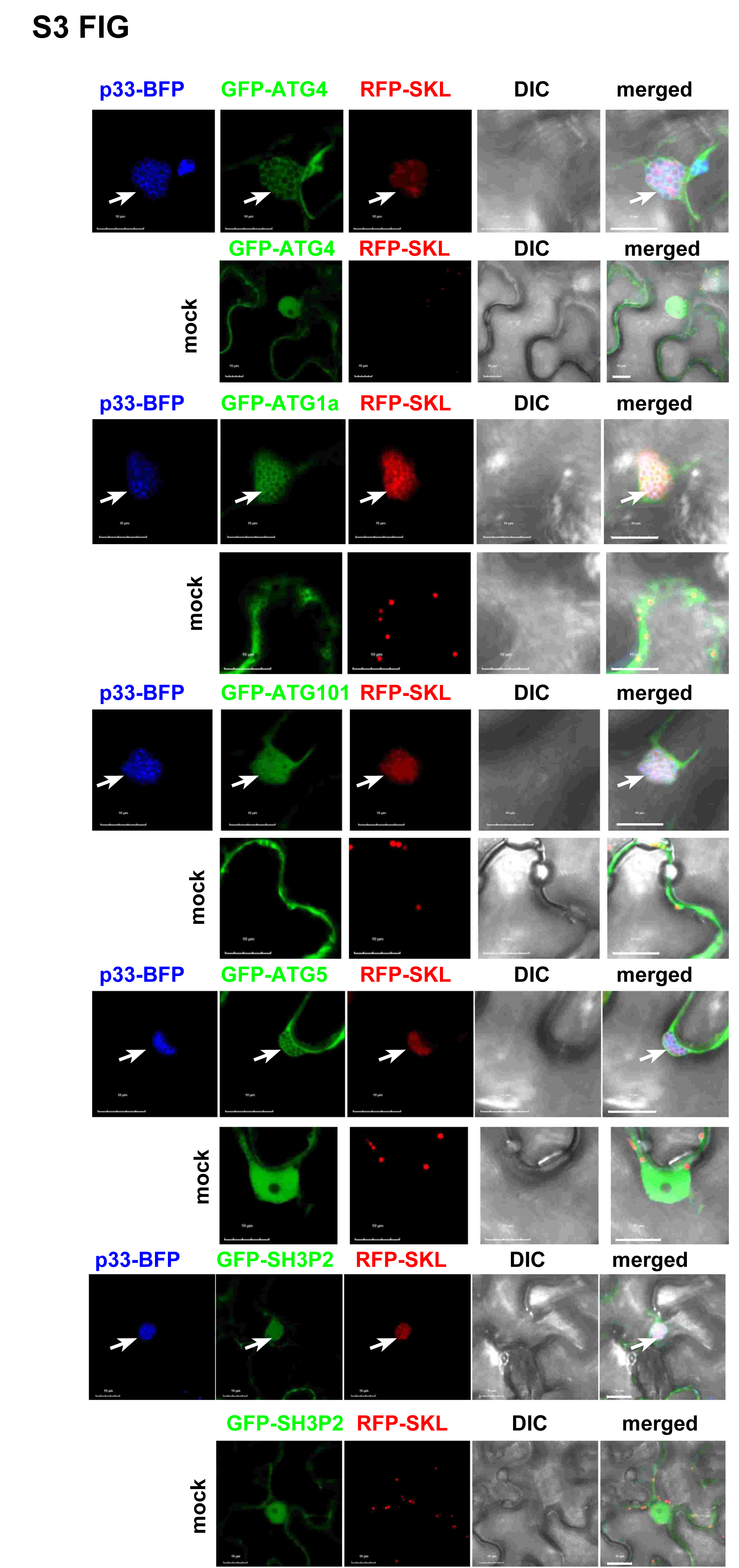

Supplement: S3 Fig — Confocal microscopy images show co-localization of TBSV p33-BFP replication protein and ATG proteins (GFP-AtATG4, GFP-NbATG5, GFP-NbATG1a, GFP-NbATG101, and GFP-AtSH3P2) within VROs consisting of clustered peroxisomes, marked by RFP-SKL peroxisomal matrix marker in N. benthamiana leaves. Control experiments included the localization of the above ATG proteins in the absence of TBSV p33-BFP replication protein. The expression of these proteins, driven by the 35S promoter, was achieved through co-agroinfiltration into N. benthamiana leaves. Scale bars represent 10 μm. Each experiment was repeated. (TIF) [file ppat.1012085.s003.tif]

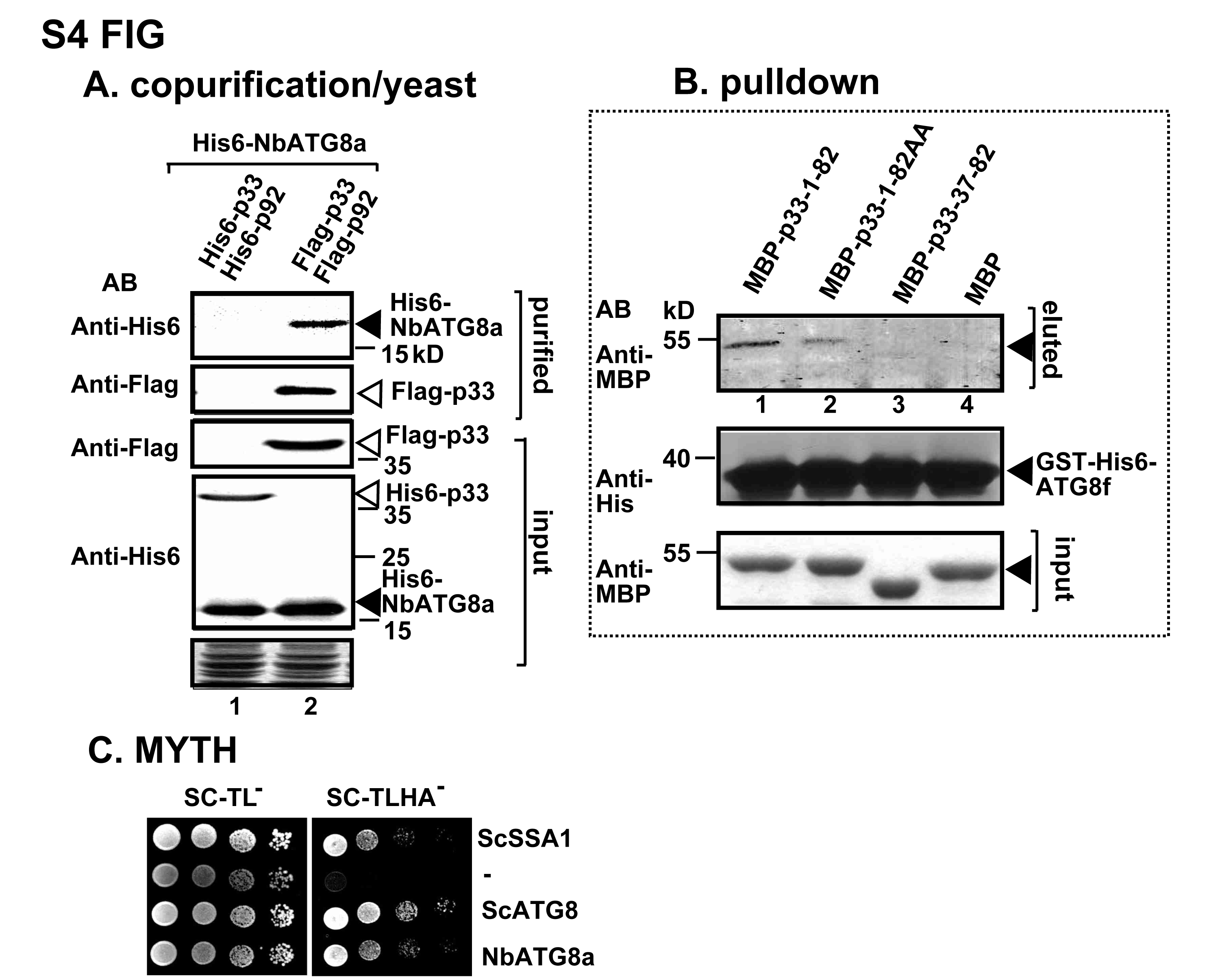

Supplement: S4 Fig — (A) Co-purification of His6-NbATG8a with TBSV Flag-p33 and Flag-p92pol replication proteins from subcellular membranes of yeast. Top two panels: western blot analysis of co-purified His6-NbATG8a detected with anti-His antibody, while Flag-p33 was detected with anti-Flag antibody. The negative control was from yeast expressing His6-p33 purified on a Flag-affinity column (lane 1). Samples were cross-linked with formaldehyde. Bottom two panels: western blot of input His6-NbATG8a and Flag-p33 in the total yeast extracts. (B) Pulldown assay including GST-His6-ATG8f and the MBP-tagged TBSV p33 replication protein. Note that we used the soluble N-terminal region (1–82 aa) of TBSV p33, which contains the predicted AIM1 motif (NIFQLV). The F and V amino acids were mutated to As to eliminate the canonical AIM1 in p33-1-82AA (S4B Fig, lane 2). Top panel: western blot analysis of the eluted MBP-p33 protein was performed with anti-MBP antibody. The negative control was the MBP (lane 4). Middle panel: Western blot analysis of the eluted GST-His6-ATG8f from the GST column. Bottom panels: Coomassie-blue stained SDS-PAGE of affinity-purified MBP-p33 proteins and MBP from E. coli. (C) The split ubiquitin-based MYTH assay was used to test binding between either GST-His6-ScATG8 or GST-His6-NbATG8a and TBSV p33 protein in yeast. The bait p33 was co-expressed with the shown prey proteins. The bait p33 and the empty prey vector (NubG) were used as negative controls, and the bait p33 and ScSSA1 as a positive control, respectively. The right panel shows the interactions, whereas the left panel demonstrates that comparable amounts of yeasts were used for these experiments. (TIF) [file ppat.1012085.s004.tif]

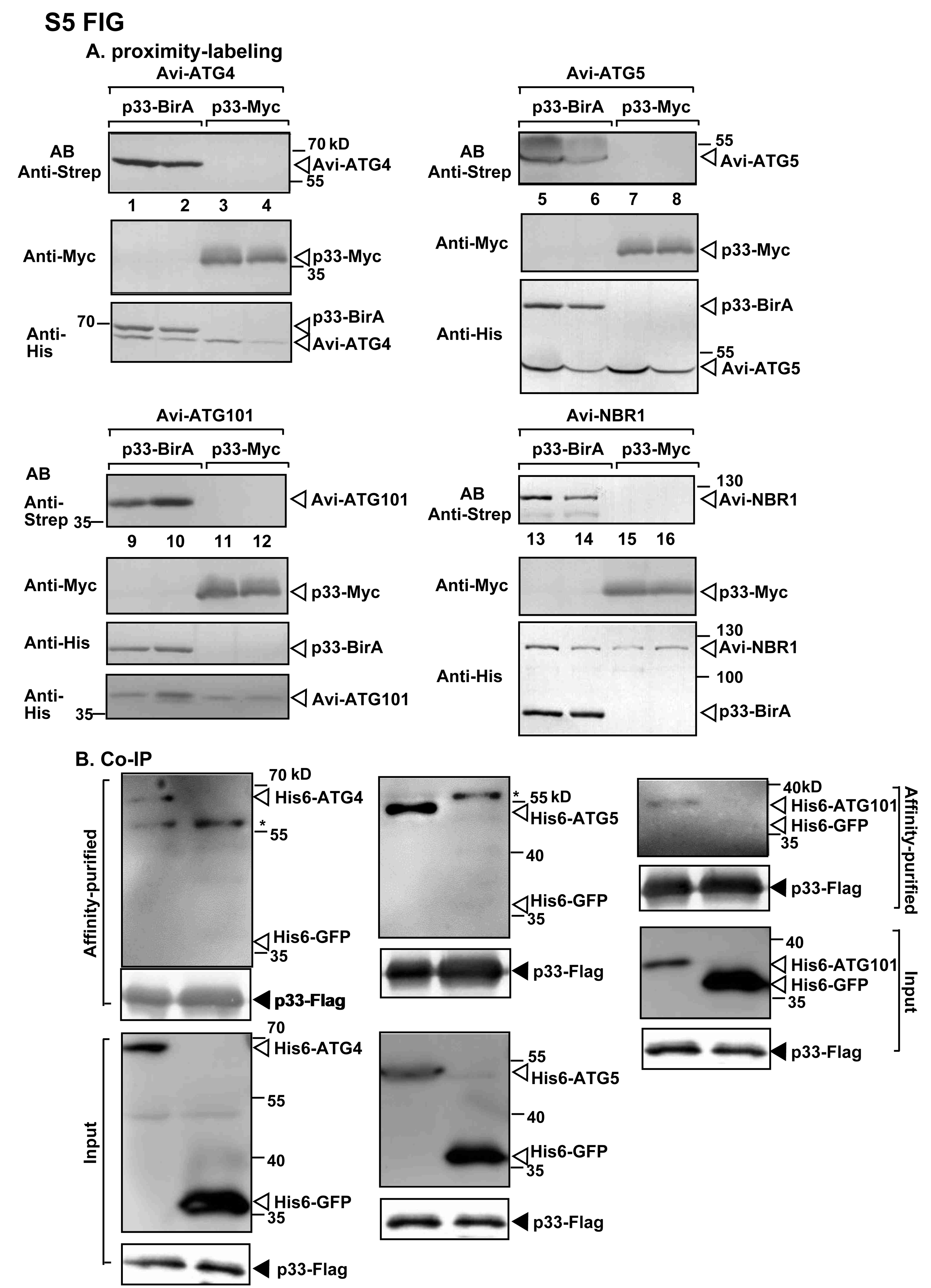

Supplement: S5 Fig — (A) Protein proximity-labeling was performed with biotin in planta. N. benthamiana leaves were agroinfiltrated to express p33 replication protein, which was fused to BirA biotin ligase, and Avi-tagged ATG proteins (Avi-ATG4, Avi-ATG5, Avi-ATG101 and Avi-NBR1). Biotin treatment lasted for 40 min. The image shows the western blot analysis of the biotinylated Avi-ATG proteins and Avi-NBR1 detected with streptavidin-conjugated AP in total protein extracts. The experiment was repeated. (B) Co-purification of ATG proteins (His6-ATG4, His6-ATG5, or His6-ATG101) with TBSV Flag-p33 replication protein from N. benthamiana plants. Top two panels: western blot analysis of co-purified His6-ATG proteins detected with anti-His antibody, whereas Flag-p33 was detected with anti-Flag antibody. Bottom panel: western blot of total His6-ATG proteins in the total protein extracts. (TIF) [file ppat.1012085.s005.tif]

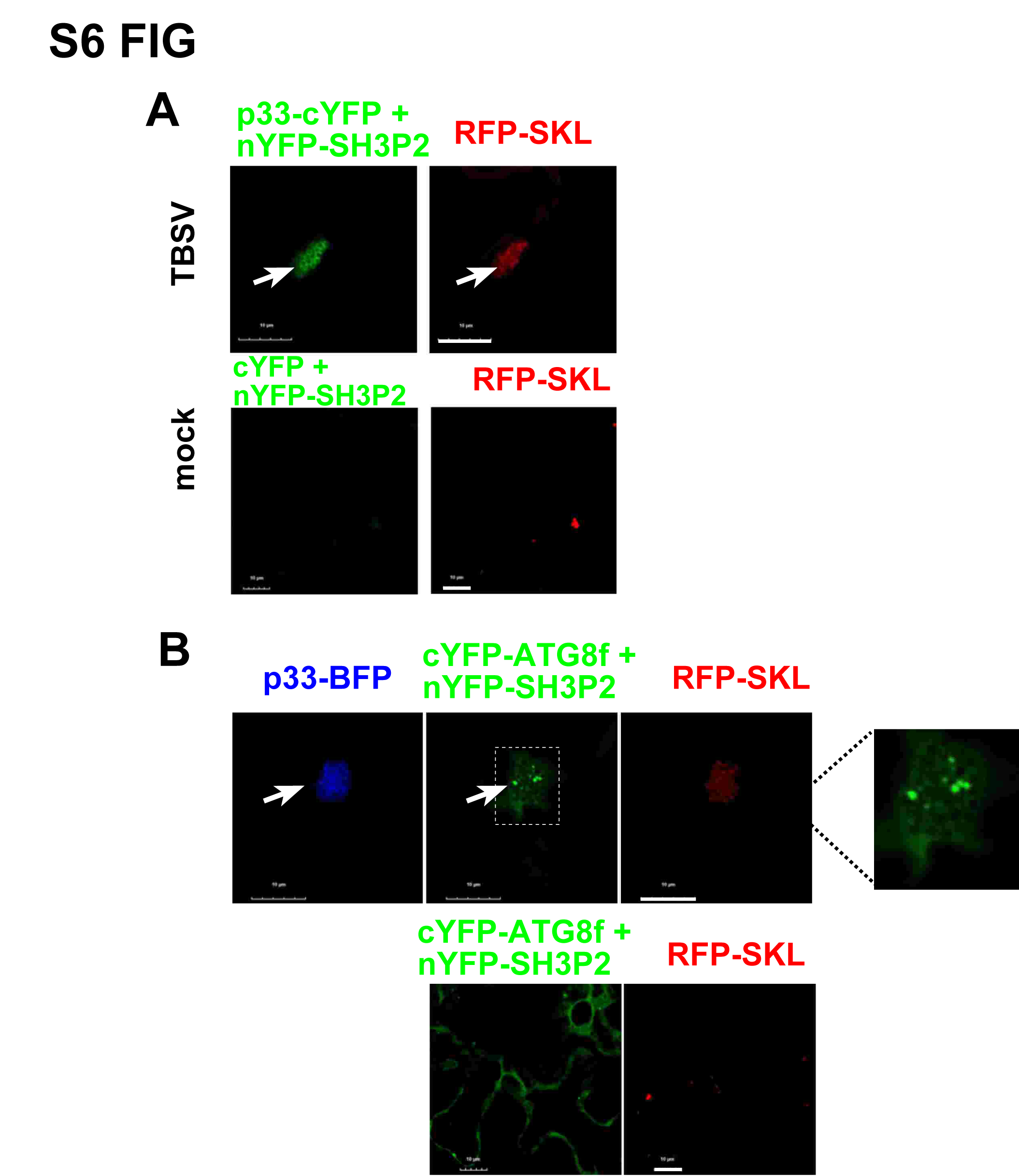

Supplement: S6 Fig — (A) Interaction between TBSV p33-cYFP replication protein and the nYFP-SH3P2 protein was detected by BiFC. The merged images show the co-localization of RFP-SKL with the BiFC signals, indicating that the interaction between p33 replication protein and SH3P2 occurs in VROs in clustered peroxisomal membranes. Scale bars represent 10 μm. (B) BiFC assay was conducted to demonstrate the interaction between nYFP-SH3P2 and cYFP-ATG8f proteins within the p33-BFP-positive VROs. The expression of proteins was achieved via co-agroinfiltration into N. benthamiana leaves. Scale bars represent 10 μm. Each experiment was repeated three times. (TIF) [file ppat.1012085.s006.tif]

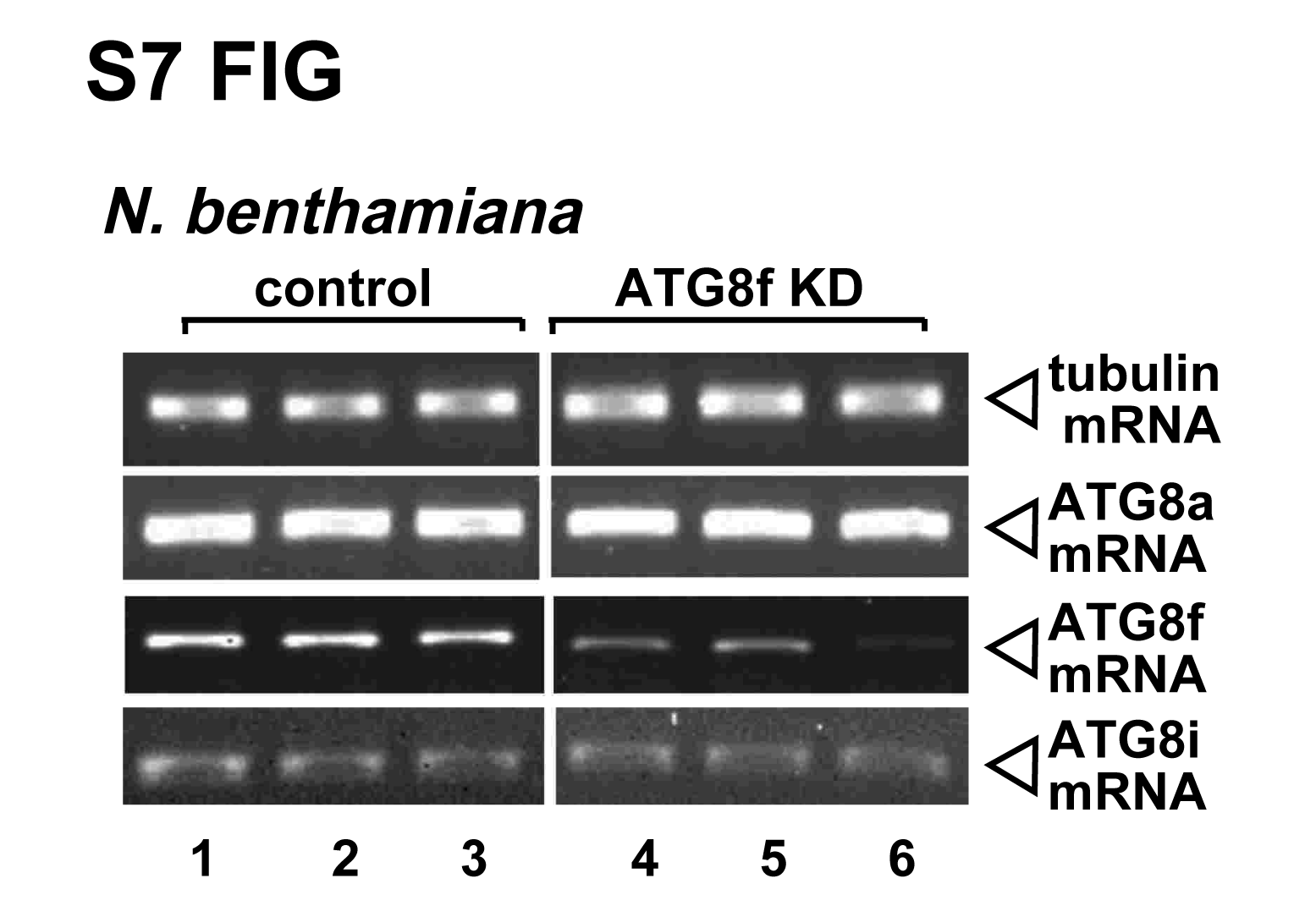

Supplement: S7 Fig — The semi-quantitative RT-PCR analysis was conducted on the same set of plant samples to assess the effectiveness of ATG8f silencing. The second panel shows comparable mRNA levels of ATG8a and bottom panel for ATG8i, indicating selective gene silencing of ATG8f. Top panel: The RT-PCR analysis of tubulin mRNA level in the ATG8f-silenced (lanes 4–6) and control (lanes 1–3) plants. The panels were from the same gels, respectively. The experiment was repeated. (TIF) [file ppat.1012085.s007.tif]

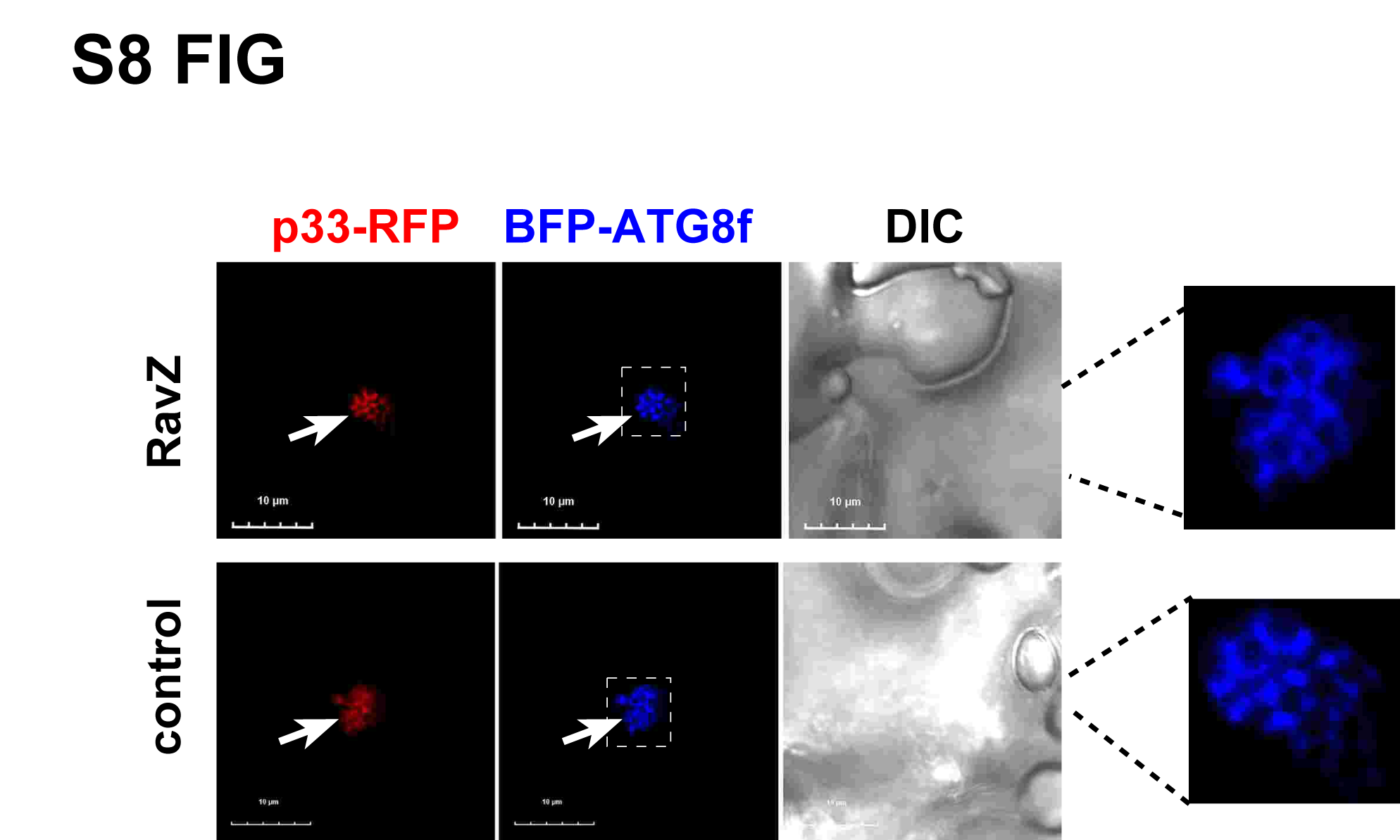

Supplement: S8 Fig — Confocal microscopy images show co-localization of RFP-ATG8f and p33-BFP in N. benthamiana cells. The leaves either expressed GFP-RavZ effector (top panel), or pGD vector as control (bottom panel). The VROs are marked with arrows. See further details in Fig 6B. The experiment was repeated. (TIF) [file ppat.1012085.s008.tif]

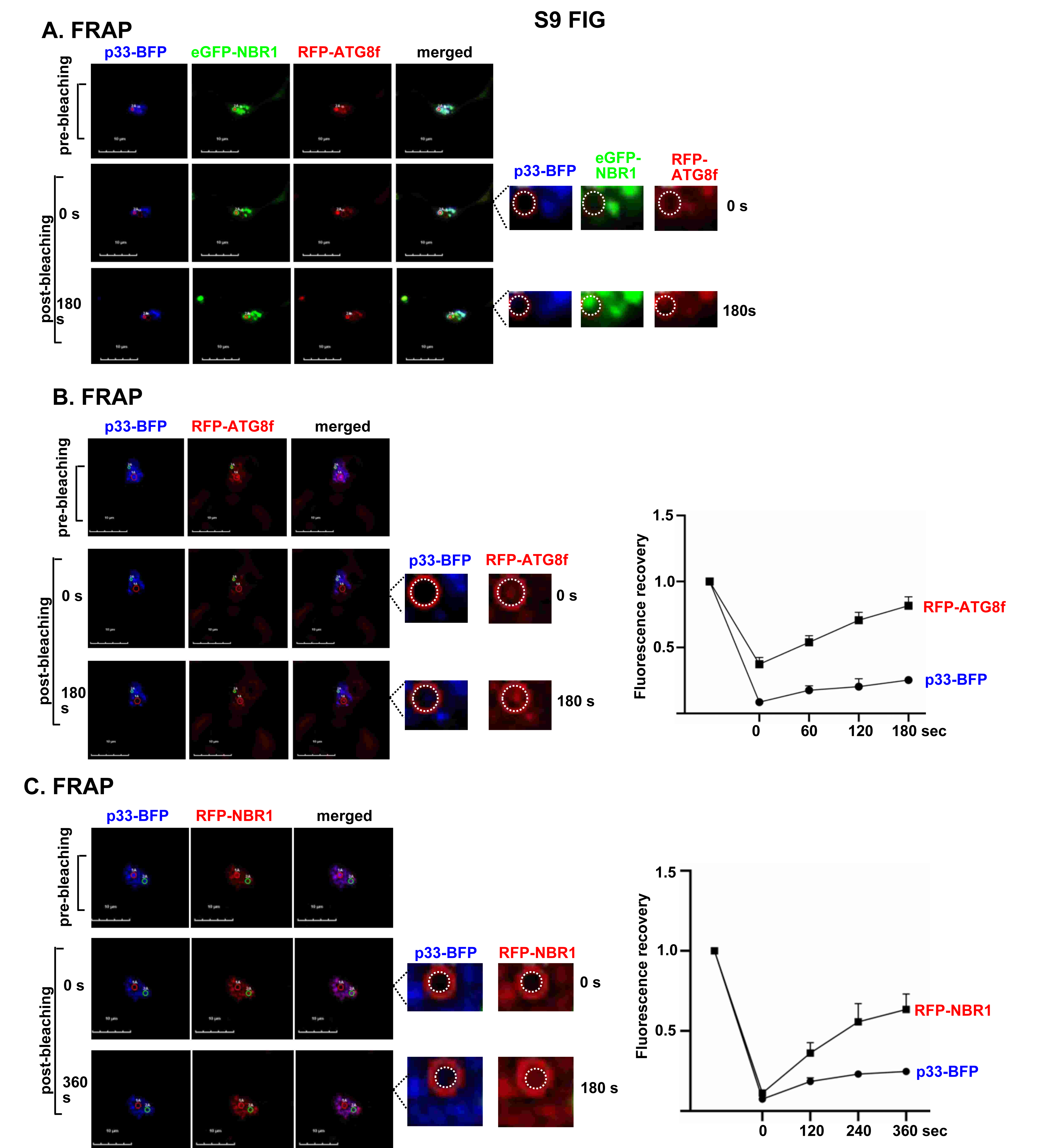

Supplement: S9 Fig — (A) Agroinfiltrated N. benthamiana leaves co-expressing p33-BFP, eGFP-NBR1 and RFP-ATG8f were used in fluorescence recovery after photobleaching (FRAP) assay. Confocal images were taken before and after photobleaching for 180 sec. Time ‘0 s’ indicates the time of photobleaching. Note that we selected a large punctate structure for photobleaching. Scale bars represent 10 μm. (B-C) Agroinfiltrated N. benthamiana leaves co-expressing p33-BFP and RFP-ATG8f (B) or p33-BFP and RFP-NBR1 (C) were used in a FRAP assay. Confocal images were taken before and after photobleaching for 180–360 sec. Time ‘0 s’ indicates the time of photobleaching. Scale bars represent 10 μm. Quantification of FRAP signals of p33-BFP, RFP-ATG8f and RFP-NBR1 in the photobleached area was done at the indicated time points after photobleaching. Confocal images of four individual VROs were taken. Scale bars represent 10 μm. Each experiment was repeated three times. (TIF) [file ppat.1012085.s009.tif]

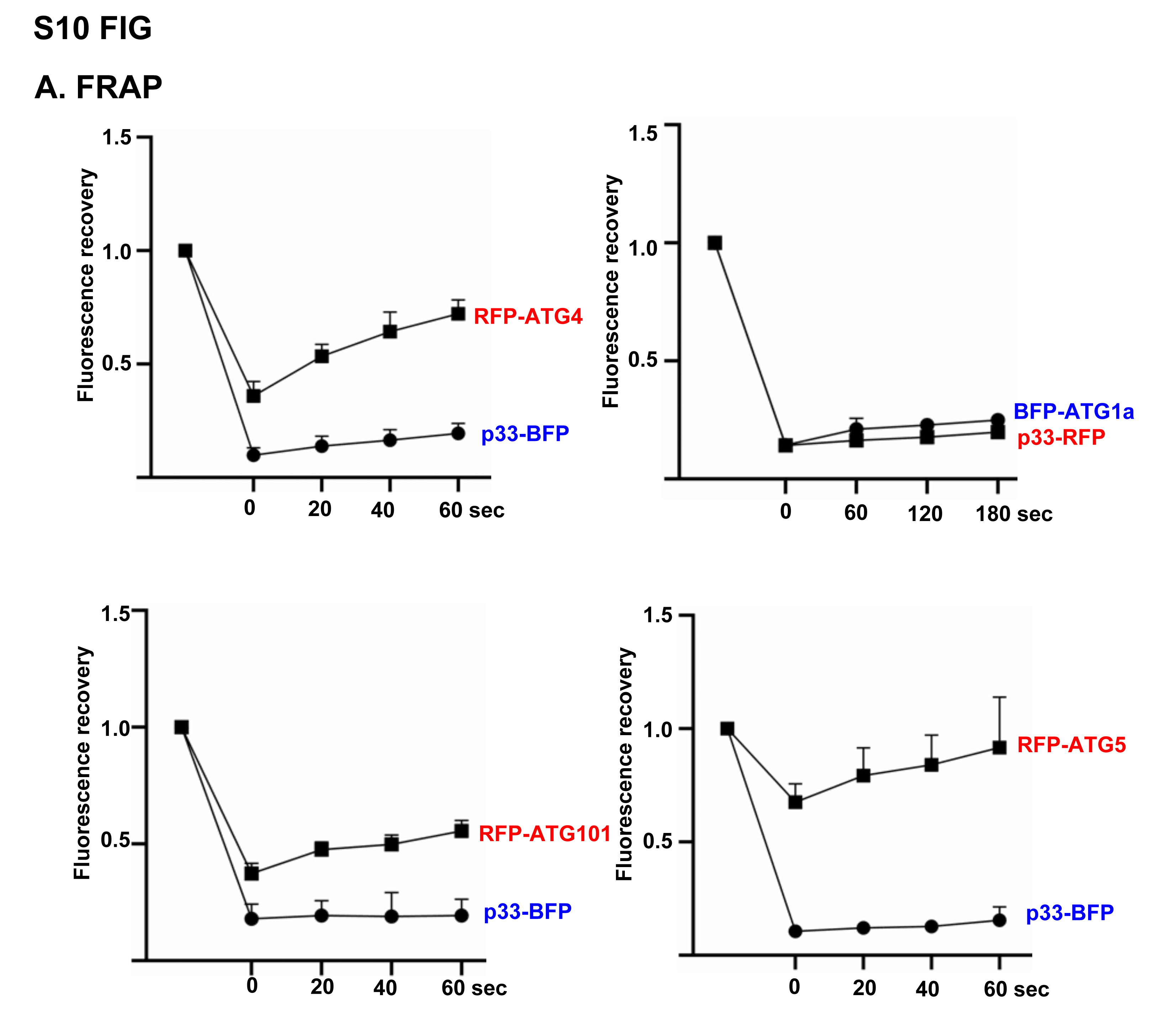

Supplement: S10 Fig — (A) Agroinfiltrated N. benthamiana leaves co-expressing p33-BFP, and one of following: RFP-ATG4, BFP-ATG1a, RFP-ATG101 and RFP-ATG5 were used in FRAP assays. Confocal images were taken before and after photobleaching for 60–180 sec. Time ‘0 s’ indicates the time of photobleaching. Quantification of FRAP signals of p33-BFP, RFP-ATG4, BFP-ATG1a, RFP-ATG101 and RFP-ATG5 in the photobleached area was done at the indicated time points after photobleaching. Confocal images of four individual VROs were taken. Each experiment was repeated three times. (TIF) [file ppat.1012085.s010.tif]
