# Supplementary material for: Subversion of selective autophagy for the biogenesis of tombusvirus replication organelles inhibits autophagy
Source: PLoS Pathog. 2024 Mar 14;20(3):e1012085. doi: 10.1371/journal.ppat.1012085 (PMC10965100; doi:10.1371/journal.ppat.1012085)
Supplement: S1 Table — (DOCX) [file ppat.1012085.s011.docx]

**S1 Table**

| Plasmids Constructed in This Study | | | | | | |
| --- | --- | --- | --- | --- | --- | --- |
| No. | Plasmid name | insert source | insert digestion sites | primers for insert amplification | vector source | vector digestion sites |
| No. 1 | pMal-T33-N82aa | pGD-35S-T33-BFP | BglII and XhoI | #4000 and #5710 | pMal-EV | BamHI and SalI |
| No. 2 | pMal-T33-N82-F32A/V35A | pGD-nYFP-T33-F32A/V35A | BglII and XhoI | #4000 and #5710 | pMal-EV | BamHI and SalI |
| No. 3 | pMal-T33-N37-82 | pGD-nYFP-T33-F32A/V35A | BglII and XhoI | #5707 and #5710 | pMal-EV | BamHI and SalI |
| No. 4 | pGD-YFP-3XHA-ATG8f | *N. benthamiana* cDNA | BamHI and XhoI | #8554 and #8555 | pGD-YFP | BamHI and SalI |
| No. 5 | pGD-eGFP-His-BirA | pGD-2X-35s-eGFP | XhoI and SalI | #8420 and #8421 | HpESC-Gal-BirA-Hisp33/Gal-DI72 | XhoI and SAP |
| No. 6 | pGEX-His-Atg8f | *N. benthamiana* cDNA | BamHI and XhoI | #8554 and #8555 | pGEX | BamHI and SalI |
