# Supplementary material for: Subversion of selective autophagy for the biogenesis of tombusvirus replication organelles inhibits autophagy
Source: PLoS Pathog. 2024 Mar 14;20(3):e1012085. doi: 10.1371/journal.ppat.1012085 (PMC10965100; doi:10.1371/journal.ppat.1012085)
Supplement: S2 Table — (DOCX) [file ppat.1012085.s012.docx]

**S2 Table**

| Plasmids Described in Previous Study | | |
| --- | --- | --- |
|  | Plasmid name | Source |
| No. 1 | pGD-nYFP-VPS34 | [1] Feng et al., 2019 |
| No. 2 | pEsc-Vps34-3xHA | [1] Feng et al., 2019 |
| No. 3 | pGBK-His-p33-DI72 | [2] Barajas et al., 2009 |
| No. 4 | pGAD-His-p92 | [2] Barajas et al., 2009 |
| No. 5 | pGBK-Flag-p33-DI72 | [2] Barajas et al., 2009 |
| No. 6 | pGAD-Flag-p92 | [2] Barajas et al., 2009 |

[1]Feng Z, Xu K, Kovalev N, Nagy PD. Recruitment of Vps34 PI3K and enrichment of PI3P phosphoinositide in the viral replication compartment is crucial for replication of a positive-strand RNA virus. PLoS Pathog. 2019;15(1):e1007530. Epub 2019/01/10. doi: 10.1371/journal.ppat.1007530. PubMed PMID: 30625229; PubMed Central PMCID: PMCPMC6342326.

[2] Barajas, D., Li, Z., & Nagy, P. D. (2009). The Nedd4-type Rsp5p ubiquitin ligase inhibits tombusvirus replication by regulating degradation of the p92 replication protein and decreasing the activity of the tombusvirus replicase. *Journal of virology*, *83*(22), 11751-11764.
