# Supplementary material for: Subversion of selective autophagy for the biogenesis of tombusvirus replication organelles inhibits autophagy
Source: PLoS Pathog. 2024 Mar 14;20(3):e1012085. doi: 10.1371/journal.ppat.1012085 (PMC10965100; doi:10.1371/journal.ppat.1012085)
Supplement: S3 Table — (DOCX) [file ppat.1012085.s013.docx]

**S3 Table**

| **Primers used in this study** | | |
| --- | --- | --- |
| No. | Name | Sequence (5' to 3') |
| 1 | #6532/eGFP/F/BglII | GGAAGATCTATGGTGAGCAAGGGCGAG |
| 2 | #8875/TBSV P33/F32A/V35A/R | TCTCAGAACCACACGACACGCCAATTGAGCGATGTTCACCGGAACTGT |
| 3 | #8874/TBSV P33/F32A/V35A/F | GCTCAATTGGCGTGTCGTGTGGTTCTGAGA |
| 4 | #810/TBSV P33/XhoI/R | GGAGCTCGAGCTATTTGACACCCAGGGAC |
| 5 | #4000/TBSV P33/BglII/F | CCAGAGATCTATGGAGACCATCAAGAGAATG |
| 6 | #1593/TBSV P33/stop/XhoI/R | CGGCTCGAGCTATTTGACACCCAGGGACTCCTGT |
| 7 | #5710/TBSV P33/82aa/XhoI/R | CCGCTCGAGTTAATCACCCCTCTGCCGTC |
| 8 | #5707/TBSV P33/37aa/BglII/F | GGAAGATCTCGTGTGGTTCTGAGATACATGAG |
